# Phylogenomic and metabolomic approaches to provide insight for species delimitation of cultivated *Ferula* species in Xinjiang, China

**DOI:** 10.3389/fpls.2026.1754844

**Published:** 2026-04-27

**Authors:** Hafiz Muhammad Wariss, Lei Yang, Jinxin Gu, Danhui Liu, Wenjun Li

**Affiliations:** 1 State Key Laboratory of Ecological Safety and Sustainable Development in Arid Lands, Xinjiang Institute of Ecology and Geography, Chinese Academy of Sciences, Urumqi, China; 2 Xinjiang Key Laboratory of Biodiversity Conservation and Application in Arid Lands, Xinjiang Institute of Ecology and Geography, Chinese Academy of Sciences, Urumqi, China; 3 China-Tajikistan Belt and Road Joint Laboratory on Biodiversity Conservation and Sustainable Use, Xinjiang Institute of Ecology and Geography, Chinese Academy of Sciences, Urumqi, China; 4 Department of Botany, University of Sargodha, Sargodha, Punjab, Pakistan; 5 The Specimen Museum of Xinjiang Institute of Ecology and Geography, Chinese Academy of Sciences, Urumqi, China; 6 College of Resources and Environment, University of Chinese Academy of Sciences, Beijing, China

**Keywords:** authentication, *Ferula*, metabolomics, multivariate analysis, phylogenomics

## Abstract

The genus *Ferula* is an important medicinal group within the Apiaceae family, valued globally for its health and culinary uses. Recently, it has gained significant attention because of its cultivation and use to meet growing demands for food and medicine. However, in the Xinjiang production region, farmers often cultivate multiple *Ferula* species together. In the market, leaves from different species are frequently bundled and sold, leading to inconsistent product quality. This issue greatly reduces the economic value of *Ferula* as a food resource and affects the efficacy and safety of its clinical applications. Correctly identifying species is essential for the sustainable use and conservation of these plants. Traditional methods mainly rely on floral and fruit traits, but the monocarpic or polycarpic nature of these plants makes species identification difficult, especially since flowering or fruiting specimens are hard to obtain in cultivated conditions. In this study, leaf samples from 30 *Ferula* specimens collected from ten cultivated locations in Xinjiang were analyzed using phylogenetic methods and ultraperformance liquid chromatography coupled with tandem mass spectrometry (UPLC-MS/MS) to provide insight for species identification and discrimination. Phylogenomic analysis based on the complete plastome was used to clarify relationships among species. The plastome super-barcoding results showed that cultivated *Ferula* species clustered into three distinct clades: *F. sinkiangensis*, *F. teterrima*, and *F. fukanensis*. Untargeted metabolomic profiling detected 7,169 metabolites, and multivariate analyses (PCA, PLS-DA and HCA) revealed clear chemotaxonomic separation among the three species, with clustering patterns consistent with the phylogenetic tree. Our findings demonstrate that the integration of plastome super-barcoding with metabolomic fingerprinting offers a robust strategy to species discrimination in cultivated *Ferula*. This combined approach provides complementary chemotaxonomic evidence for differentiating species, with direct implications for ensuring appropriate use in distinct culinary, medicinal, and pharmaceutical applications.

## 1 Introduction

Plants have long served as a vital source of pharmacologically active compounds that support and maintain human health (Shaw et al., 2025). For decades, industries have heavily relied on naturally harvested resources, such as *Polygonati Rhizoma*, as raw materials (Zhu et al., 2014; Guo et al., 2022). However, the growing demand for these materials has led to the overexploitation of natural populations, causing dramatic declines in their availability (Li et al., 2020; Guo et al., 2022). Conserving wild plant populations is substantially crucial and urgently required in order to avoid their extinction, which is extensively used as traditional medicine (Bakhshipour et al., 2019). To address these deficiencies, commercial cultivation programs have been implemented, such as those for medicinal *Polygonatum* in China since 2000, which have alleviated raw material dearth while generating economic benefits and reducing rural poverty (Shen et al., 2019; Guo et al., 2022). The cultivation of medicinal plants has become increasingly significant in recent years due to their essential role in the pharmaceutical and food industries (Guo et al., 2022). Similarly, the cultivation of *Ferula* species, a valuable medicinal plant, in Xinjiang holds great potential. This initiative could address the increasing demand for its medicinal parts while also creating economic opportunities.

The genus *Ferula* L. (Apiaceae), comprising approximately 180–185 species, is widely distributed from the Mediterranean region to Central Asia (Mohammadhosseini et al., 2019; Salehi et al., 2019; Wariss et al., 2025). Morphologically, the genus is characterized as perennial mono- and polycarpic herbs with thick, tall stems and trisected rosette leaves. Its umbels are arranged in panicles, typically without bracteoles. *Ferula* is recognized as one of the most taxonomically complex genera in the Apiaceae family, due to the similar morphologies of its species (Ajani et al., 2008; Yang et al., 2022). *Ferula* is distinguished by the presence of oleo-gum-resins in its roots and stems, which impart a distinct bitter taste and a pungent odor due to high sulfur-containing compounds and additional phytochemicals (Jiang et al., 2023; Khayat et al., 2023). Historically, *Ferula* has held significant value in traditional practices, serving as a versatile herbal medicine, a flavoring agent, and a seasoning in culinary preparations (Boghrati and Iranshahi, 2019; Wang et al., 2023a, Wang et al., 2023b; Bagheri et al., 2024). The medicinal significance of *Ferula* is rooted in its extensive use for treating various ailments, including skin infections, diarrhea, intestinal parasites, influenza, and malaria (Wang et al., 2020; Sonigra and Meena, 2021). Numerous bioactive metabolites have been isolated from *Ferula* species, such as kellerin, umbelliprenin, galbanic acid, and sinkiangenorin, etc., exhibiting a wide array of biological activities, including antiviral, anti-inflammatory, anticancer, antidiabetic, antibacterial, etc., highlighting the immense therapeutic potential of *Ferula* species (Boghrati and Iranshahi, 2019; Mohammadhosseini et al., 2019).

**Figure 1:**
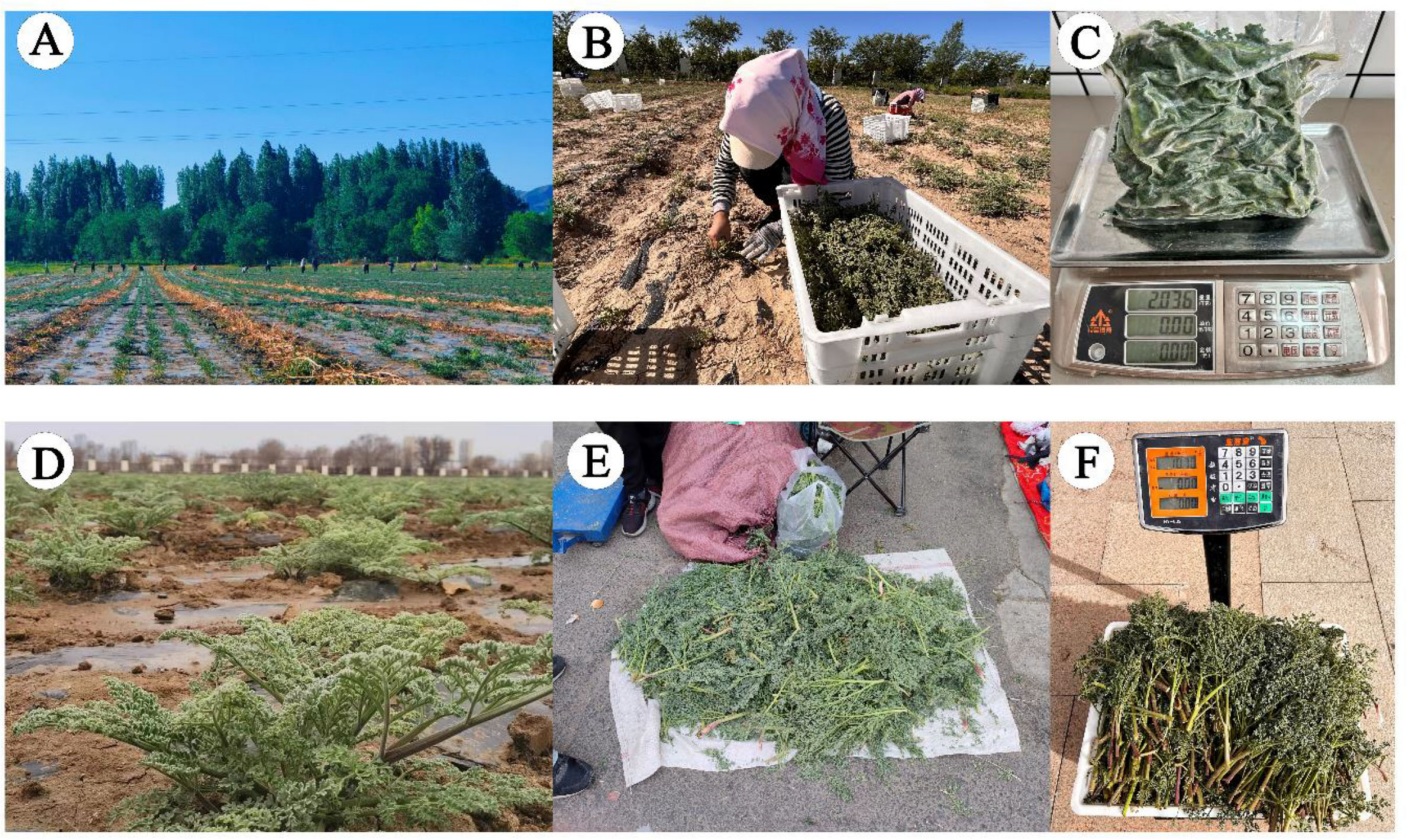
Cultivation and utilization of *Ferula* species in Xinjiang, China. **(A)** Field cultivation of different *Ferula* species; **(B)** Harvesting of aerial parts and young leaves; **(C)** post-harvest packing and weighing; **(D)** young plants cultivated for culinary and medicinal purposes; and **(E, F)** Fresh leaves sold at local bazaar in Urumqi, Xinjiang, China.

In China, the genus *Ferula*, known as “a wei shu”, includes 31 species, 25 of which are endemic to the Xinjiang (Jiang et al., 2023; Wang et al., 2023c; Wu et al., 2025). Among this diversity, several *Ferula* species native to Xinjiang serve as regional substitutes for the traditional Chinese medicine (TCM) “a wei” (*Ferula assafoetida* L.) (Wang et al., 2018; Zhang et al., 2019; Li et al., 2020). *Ferula*, has a documented history dating back to the Tang Dynasty classic “Tang Bencao” (AD 659) and is listed in the Pharmacopoeia of the People’s Republic of China (Zhang et al., 2020a). It is also recognized in the Uyghur Medicine Criteria and traditionally used to treat rheumatoid arthritis and stomach disorders (Yang et al., 2006; Wang et al., 2020; Shoaib et al., 2023; Wang et al., 2023d). *Ferula*, a genus of significant ethnobotanical and economic importance in Xinjiang, has historically been used as a vegetable and a medicinal herb. Over time, cultivation efforts emerged in local communities, and *Ferula* species were introduced from the wild into home gardens for culinary, ornamental, and therapeutic purposes. However, the plant’s rising commercial value has spurred large-scale cultivation by companies and farmers, who often collect seeds indiscriminately from wild populations, local gatherers, or informal networks without knowing or prioritizing species-specific identification. Commercial cultivation intensified due to the plant’s high market value, driven by demand for its leaves and roots in food and traditional medicine, yet this practice introduced significant ambiguity in *Ferula* species identification. The fresh aerial parts, particularly the leaves, are sold at a rate of $9 per kilogram and are commonly used in vegetable dishes, curries, and traditional local cuisine (**Figure 1**). Growers and sellers often label products generically as “*Ferula*” or “awei” (its Chinese name), obscuring critical distinctions between species such as *Ferula sinkiangensis K.M.Shen*, *F. ferulaeoides* Korovin, and others, which vary in chemical composition and therapeutic efficacy. This ambiguity has fragmented the understanding of cultivated species and raised concerns about the consistency, safety, and effectiveness of *Ferula*-based products.

Taxonomic delimitation within *Ferula* has traditionally depended on floral and fruit morphology, which are often difficult to access in cultivated or immature specimens. This creates a major challenge for applied contexts, where cultivation and market circulation focus on vegetative parts (e.g., leaves) for culinary or medicinal purposes. Additionally, many *Ferula* species are monocarpic (or polycarpic with delayed, irregular flowering), making reproductive structures unavailable for years or entirely absent in harvested material. Despite this, harvested materials from different species are sold in markets under the broad name “*Ferula*” and are valued for their medicinal properties, leading to inconsistent and highly variable product quality. Therefore, resolving this taxonomic uncertainty is essential for standardizing cultivation practices, conserving wild genetic resources, and ensuring the quality, integrity, and reputation of *Ferula* products in local and international markets.

Species identification has traditionally been a challenging task when relying solely on morphological evidence (Duminil and Di Michele, 2009). However, modern methods that integrate morphological data with genetic analysis and chemotaxonomy, including DNA sequencing and metabolomics, have emerged as effective approaches for molecular authentication and distinguishing medicinal plants (Duan et al., 2012; Gallon et al., 2018; Chervin et al., 2019; Negrin et al., 2019; Zhao et al., 2019; Cheng et al., 2020; Fayek et al., 2021);. Advancements in high-throughput sequencing technologies have led to the use of genomic data, such as complete plastid genome (plastome) sequences (Chong et al., 2022; Shen et al., 2024), which are now recommended as “super-barcodes” to address challenges in species identification and delimitation that standard DNA barcodes cannot resolve (Nock et al., 2011; Dodsworth, 2015). This advancement greatly improves the resolution of species identification and the authentication of traditional medicinal plants (Alberts and Meyer, 2022; Wang et al., 2023e; He et al., 2024). Plant secondary metabolites play a crucial role in distinguishing morphologically similar species (Nguyen et al., 2016). The phytochemical diversity inherent in these metabolites provides a foundation for understanding the evolution and classification of plant species (Shen et al., 2022). Metabolomics, as a widely adopted method, enables comprehensive profiling and comparison of phytochemical compositions across species (Li et al., 2022). This approach, when applied in chemotaxonomy, offers both qualitative and quantitative insights into a vast array of metabolites, significantly enhancing the accuracy of species identification and quality assessment (Masson et al., 2014; Zhang et al., 2023).

In the current work, we hypothesize that an integrated approach, combining phylogenomic analysis based on complete plastomes with untargeted metabolomic profiling, will provide a robust and reliable method for accurately delineating and authenticating the cultivated *Ferula* species in Xinjiang, overcoming the limitations of traditional methods. Therefore, the primary objectives of this study was: (1) to construct a phylogenomic framework for cultivated Xinjiang *Ferula* using complete plastome sequences; (2) to profile the metabolomic diversity of these accessions using UPLC-MS/MS; and (3) to integrate phylogenetic and chemotaxonomic data to establish a definitive method for species identification. The results of this research will serve as a foundation for the accurate authentication of cultivated *Ferula* species, ensuring the integrity of medicinal materials, with potential applications in medicinal and sustainable utilization of this valuable medicinal resource.

## 2 Materials and methods

### 2.1 Plant materials

In this study, filed visits were conducted to ten agricultural farms in Xinjiang, China, to collect fresh leaf material of cultivated *Ferula* species. Given that the primary objective of this study was species authentication, the sampling strategy prioritized capturing interspecific diversity. From each farm, three individual plants were sampled, yielding a total of 30 fresh leaf samples collected across the ten sites. Therefore, for each species identified, we collected multiple biological replicates (distinct individuals), ensuring that metabolic variation could be assessed at the species level. Detailed information on the samples and their collection sites is provided in **Supplementary Table 1**. To minimize the confounding effects of environment and development on metabolomic profiles, strict sampling controls were implemented. All leaf tissue was harvested from plants at an identical phenological stage (pre-flowering) and using the same leaf position (Basal leaf) to minimize developmental variation. For phylogenetic analysis, leaf material from each of the 30 individual plants were collected and immediately preserved in silica gel. For non-targeted metabolomic analysis, leaves were separately collected from the same 30 individual plants, transported to the laboratory in dry ice, and stored at -80 °C until processing. All sampling was conducted in June 2024. Additionally, sample 31^st^ corresponds to a wild *F. fukanensis* specimen obtained from the herbarium. The collection and identification processes complied with institutional, national, and international guidelines (Buck and Hamilton, 2011; Gallagher et al., 2025). Voucher specimens were deposited at the Herbarium of the Xinjiang Institute of Ecology and Geography, Chinese Academy of Sciences (XJBI).

### 2.2 Morphological evaluation

All photographs were captured using a Nikon Z7II camera to record morphological features for morphometric studies. Macro images were obtained using a Laowa 25mm F2.8 2.5-5X macro lens mounted on a sliding track to ensure stability and precision in capturing fine details. The clarity of the photographs was enhanced through the depth of field stacking using Helicon Focus Pro version 6.3. This technique combined multiple images taken at varying focal depths, resulting in high-resolution composite images suitable for detailed morphological studies.

### 2.3 Phylogenomic studies

#### 2.3.1 Genomic DNA extraction, library preparation, and sequencing

Genomic DNA was extracted from silica-dried leaves using the plant genome extraction kit (DP320) from Tiangen Biochemical Technology (Beijing, China) following the manufacturer’s instructions. The quality and quantity of the extracted DNA were assessed using a NanoDrop spectrophotometer and agarose gel electrophoresis. All samples had an A260/A280 ratio between 1.8 and 2.0 and an A260/A230 ratio > 2.0, confirming high purity and minimal contamination. A total amount of 1.5 *µ*g DNA per sample was used for library preparation. Sequencing libraries were generated using Truseq Nano DNA HT Sample Preparation Kit (Illumina USA) following the manufacturer’s recommendations, and index codes were added for sample identification. Briefly, the DNA sample was fragmented by sonication (Covaris M220, 200 cycles per burst, 55 seconds treatment time) to a size of 350 bp, then the DNA fragments were end polished, A-tailed, and ligated with the full-length adapter for Illumina sequencing, followed by further PCR amplification (8 cycles). Finally, PCR products were purified (AMPure XP system) and libraries were analyzed for size distribution by Agilent2100 Bioanalyzer and quantified using real-time PCR. Paired-end sequencing (150 bp) of genomic DNA was conducted on the Illumina NovaSeq 6000 platform with an insert size of approximately 350 bp. Raw data were processed using in-house C scripts for quality control, removing unreliable reads and PCR duplicates (identical paired-end reads) to ensure reliability and reduce bias in subsequent analysis. The complete plastome sequences newly generated in this study have been deposited in GenBank under accession numbers PQ834963 to PQ834992 (a full list is provided in **Supplementary Table 1**). For subsequent phylogenetic analysis, the newly sequenced plastomes were aligned with publicly available, morphologically verified plastome sequences of *Ferula* species downloaded from GenBank (e.g., OP324724 for *F. teterrima*, MW411057 for *F. sinkiangensis*), which served as positive controls to validate our assembly and annotation pipeline.

#### 2.3.2 Genome assembly and annotation

The GetOrganelle v. 1.7.7.0 pipeline (https://github.com/Kinggerm/GetOrganelle) was used to obtain plastid-like reads by filter paired-end read (Jin et al., 2020) and SPAdes v. 3.10 plugin in the GetOrganelle pipeline was used to assemble reads (Bankevich et al., 2012). The complete circular assembly was extracted and visualized using Bandage v. 0.8.1 (Wick et al., 2015). The genomes were annotated using CpGAVAS (Liu et al., 2012) and PGA (https://github.com/quxiaojian/PGA), followed by manual checked and adjustments in Geneious v. 9.1.7 (Kearse et al., 2012) to correct putative start/stop codons and intron positions following comparisons with the reference plastome (*F. transiliensis* (Regel & Herder) Pimenov: ON324040). Finally, the plastome sequences generated in this study were submitted to GenBank for accession numbers (**Supplementary Table 1**).

### 2.3.3 Phylogenetic analysis

The 31 plastomes newly sequenced in this study were combined with 20 additional *Ferula* plastomes and two outgroup species (*Cuminum cyminum* L.*, Daucus aureus* Desf.), all of which were downloaded from the NCBI database, and employed for phylogenetic analysis. In total, 53 complete plastome sequences were initially aligned using MAFFT v.7.221 with the G-INS-I algorithm in Geneious (Katoh and Standley, 2013). The resulting alignment was subsequently trimmed in TrimAl v.1.2 (Capella-Gutierrez et al., 2009) using the ‘automated1’ parameter to remove ambiguously aligned regions, sequences of uncertain homology, and single-taxon insertions (Capella-Gutierrez et al., 2009; Chen et al., 2016). ModelFinder (Kalyaanamoorthy et al., 2017) was used to select the best-fit model of nucleotide substitution with the Bayesian Information Criterion (BIC) within Phylosuite v.1.2.3 (Zhang et al., 2020b). The Maximum Likelihood (ML) tree was inferred using IQ-TREE v. 2 (Stamatakis, 2014; Drew et al., 2017) with a GTR+I+G model and assessed with 1,000 bootstrap replicates. MrBayes v.3.2.7 (Ronquist et al., 2012) was employed to construct a Bayesian Inference (BI) phylogenetic tree utilizing the optimal DNA substitution model (GTR+F+I+G4). The Markov chain Monte Carlo (MCMC) analysis was run with two independent replicates, each consisting of four chains starting from a random tree. The chains were sampled every 1,000 generations over 20 million generations, continuing until the average standard deviation of split frequencies fell below 0.01. A burn-in of 25% was discarded to account for initial chain non-stationarity. Phylogenetic trees were visualized using FigTree v.1.4.2 (Rambaut, 2014). In addition to plastome analysis, we also extracted and analyzed the nuclear Internal Transcribed Spacer (ITS) and External Transcribed Spacer (ETS) sequences from the 31 sampled taxa. Each marker was aligned separately using MAFFT v.7.221, after which the sequences were concatenated using Phylosuite. Phylogenetic analysis of the concatenated nuclear dataset followed the same methodology as applied to the plastome data.

### 2.4 Metabolomic analysis

#### 2.4.1 Sample preparation and extraction

Metabolites extraction from *Ferula* samples was performed according to the standard protocol provided by Wuhan MetWare Biotechnology Co., Ltd (Li et al., 2023). A comprehensive quality control (QC) strategy was implemented throughout the analytical process. To monitor system background and cross-contamination, solvent blanks (70% methanol) were injected at the beginning, end, and after every 10 experimental samples. Additionally, a pooled QC sample, prepared from equal aliquots of all experimental extracts, was analyzed after every 10 experimental injections to monitor instrument stability over time. Briefly, plant samples were prepared using vacuum freeze-drying technology in a lyophilizer (Scientz-100F) for 63 hours. The dried samples were ground into a fine powder using a mixer mill (MM 400, Retsch) operating at 30 Hz for 1.5 min. To correct for potential losses during sample processing and fluctuations in instrument response, 2-chlorophenylalanine (98% purity, 1 ppm final concentration; J&K Scientific, CAS: 14091-11-3) was added as an internal standard to the extraction solvent. A total of 50 mg of powdered sample was weighed using an analytical balance (MS105DM) and extracted with 1200 *μ*L of pre-cooled (-20 °C) 70% methanolic aqueous solution containing the internal standard. The order of sample extraction and LC-MS injection was not randomized, which is a potential source of technical bias. However, the high reproducibility observed in the quality control (QC) samples interspersed throughout the analytical sequence demonstrates exceptional system stability. This indicates that non-biological technical variation was effectively controlled and had minimal impact on the identification of differential metabolites. Samples were vortexed every 30 min for 30 sec, repeated six times, and centrifuged (12,000 rpm, 3 minutes). The resulting supernatants were filtered through a 0.22 *μ*m microporous membrane and stored in vials at -80 °C before performing the ultra-performance liquid chromatography-tandem mass spectrometry (UPLC-MS/MS) analysis.

#### 2.4.2 Metabolite analysis by UPLC-MS/MS

Leaf sample extracts were analyzed using the UPLC-MS/MS system in both positive and negative ionization modes, following the methods described by Wuhan MetWare Biotechnology Co., Ltd. and as previously referenced (Li et al., 2022; Feng et al., 2024; Li et al., 2024). Both analyses utilized a Waters ACQUITY Premier coupled with an HSS T3 column (1.8 *µ*m, 2.1 mm × 100 mm) with mobile phases consisting of 0.1% formic acid in water (solvent A) and 0.1% formic acid in acetonitrile (solvent B). The gradient program was as follows: started at 5% B, increased linearly to 20% B over 2 min, then to 60% B over the next 3 min, and further increased to 99% B in 1 min, holding at 99% B for 1.5 min. The mobile phase was then returned to 5% B within 0.1 min and held for 2.4 min. The chromatographic conditions for both methods were identical, with a column temperature of 40 °C, a flow rate of 0.4 mL min^-1^, and an injection volume of 4 *µ*L.

The mass spectrometer was operated using the information-dependent acquisition (IDA) mode with Analyst TF 1.7.1 Software (Sciex, Concord, ON, Canada). The ion source conditions included ion source gas 1 (GAS1) and gas 2 (GAS2) at 50 psi, a curtain gas (CUR) at 25 psi, and a source temperature (TEM) of 550 °C. The ion spray voltage floating (ISVF) was set to +5000 V for positive mode and -4000 V for negative mode, with a declustering potential (DP) of ±60 V. The TOF MS scans were conducted over a mass range of 50–1000 Da with an accumulation time of 200 min and dynamic background subtraction enabled. For product ion scans, the mass range was set from 25–1000 Da with an accumulation time of 40 min, and a collision energy of ±30 V with a spread of 15 V. The resolution was set to UNIT, and a maximum of 18 candidate ions were monitored per cycle, with isotopes within 4 Da excluded and a mass tolerance of 50 ppm (Li et al., 2024).

#### 2.4.3 Data processing and multivariate analysis

The acquired MS files from the mass spectrometry analysis were exported in mzXML format by ProteoWizard (Holman et al., 2014). The mzXML files were processed using the XCMS R Package for peak extraction, alignment, and retention time correction. XCMS parameters included mass tolerance of ±15 ppm, and peak width range of 5–30 seconds. Peaks with a missing rate exceeding 50% in any sample group were excluded, and missing values were imputed by means of the K-nearest neighbors (KNN) (K = 5) method using R package impute 1.56.0. The peak areas were calibrated using the Support Vector Regression (SVR) method. After calibration and filtering, metabolites were identified based on the self-built MetWare database (MWDB) (Chen et al., 2013), integrating public databases, viz., PubChem (Kim et al., 2025), Metlin (Smith et al., 2005), and predictive libraries, and aligned by MetDNA2 (Zhou et al., 2022). The internal standard was used for process monitoring only and was not used for quantitative normalization of other metabolites. Finally, metabolites with an identification score above 0.5 and a QC sample coefficient of variation (CV) value below 0.5 were retained. Data from both positive (ESI^+^) and negative (ESI^-^) ion modes were merged, retaining metabolites with the highest identification scores and the lowest CV values.

To understand the similarities and differences between metabolic components among species, a multivariate analysis was conducted on mass spectrometry data obtained from UPLC-MS/MS full scan mode using the XCMS software. Multivariate analyses, including principal component analysis (PCA), hierarchical cluster analysis (HCA), and partial least squares-discriminant analysis (PLS-DA), were conducted using the statistics function in the R package prcomp (ver. 3.5.1), ComplexHeatmap (v. 1.2.1; 2.71.1009), and MetaboAnalyst R package (v 4.0, R) (www.r-project.org) respectively, using full-scan-MS acquisitions in both positive and negative ion modes. Metabolite data were normalized using unit variance (UV) scaling (Base package 4.1.2) and visualized using heatmaps generated with the R ComplexHeatmap package to gain insight into the distinct metabolic profiles among *Ferula* samples used in this study. Initially, to understand the structure of the metabolomic dataset, unsupervised PCA was performed, with relationships visualized using score plots of the first two principal components (PC1 vs. PC2). QC samples displayed a well-clustered distribution in principal component analysis (**Supplementary Figures 1**, **2**), confirming analytical system stability and data reliability throughout the acquisition process. The PLS-DA model’s performance was assessed using R^2^X, R^2^Y, and Q^2^ metrics. R^2^X and R^2^Y represent the explained variance of the X and Y matrices, respectively, while Q^2^ indicates the model’s predictive capability. Values closer to 1 signify a more robust model; Q^2^ > 0.5 denotes an effective model, and Q^2^ > 0.9 reflects an outstanding model. It is important to note that no independent external test set was used in this study. Therefore, the PLS-DA model and its derived VIP scores should be interpreted as exploratory tools for highlighting group differences and guiding biomarker selection, rather than as a validated predictive classifier for new samples. Model validity was confirmed through permutation testing (200 iterations). The p-values for R^2^Y and Q^2^ were calculated based on the frequency of random models outperforming the original model in explained variance (R^2^Y) and predictive ability (Q^2^). The PLS-DA model demonstrates strong discriminative power (R²Y = 0.57–0.59) and predictive accuracy (Q² = 0.608), validated by permutation testing (p < 0.005) in positive ion mode (**Supplementary Figures 3**, **4**). Similarly, in negative ion mode, the PLS-DA model demonstrated strong discriminative power (R²Y = 0.59, Q² = 0.69) and robustness (permutation test p < 0.005 for both Q² and R²Y) (**Supplementary Figures 5**, **6**), revealing that these findings support its use in identifying biologically relevant metabolites driving group differences. Subsequently, supervised PLS-DA was applied to enhance group discrimination among species. Metabolites contributing significantly to class separation were identified based on variable importance in projection (VIP) scores, with a threshold of VIP > 1.0 used to select discriminatory features. Model validity was confirmed via 7-fold cross-validation (CV) and permutation testing (n = 1,000 iterations, p < 0.05). Metabolites were prioritized using VIP scores (VIP > 1.0) derived from the PLS-DA model (biological replicates ≥ 3). Finally, HCA was employed to elucidate the chemotaxonomic relationships among *Ferula* species with results displayed as dendrogram-coupled heatmaps showing sample and metabolite clustering patterns.

**Figure 2:**
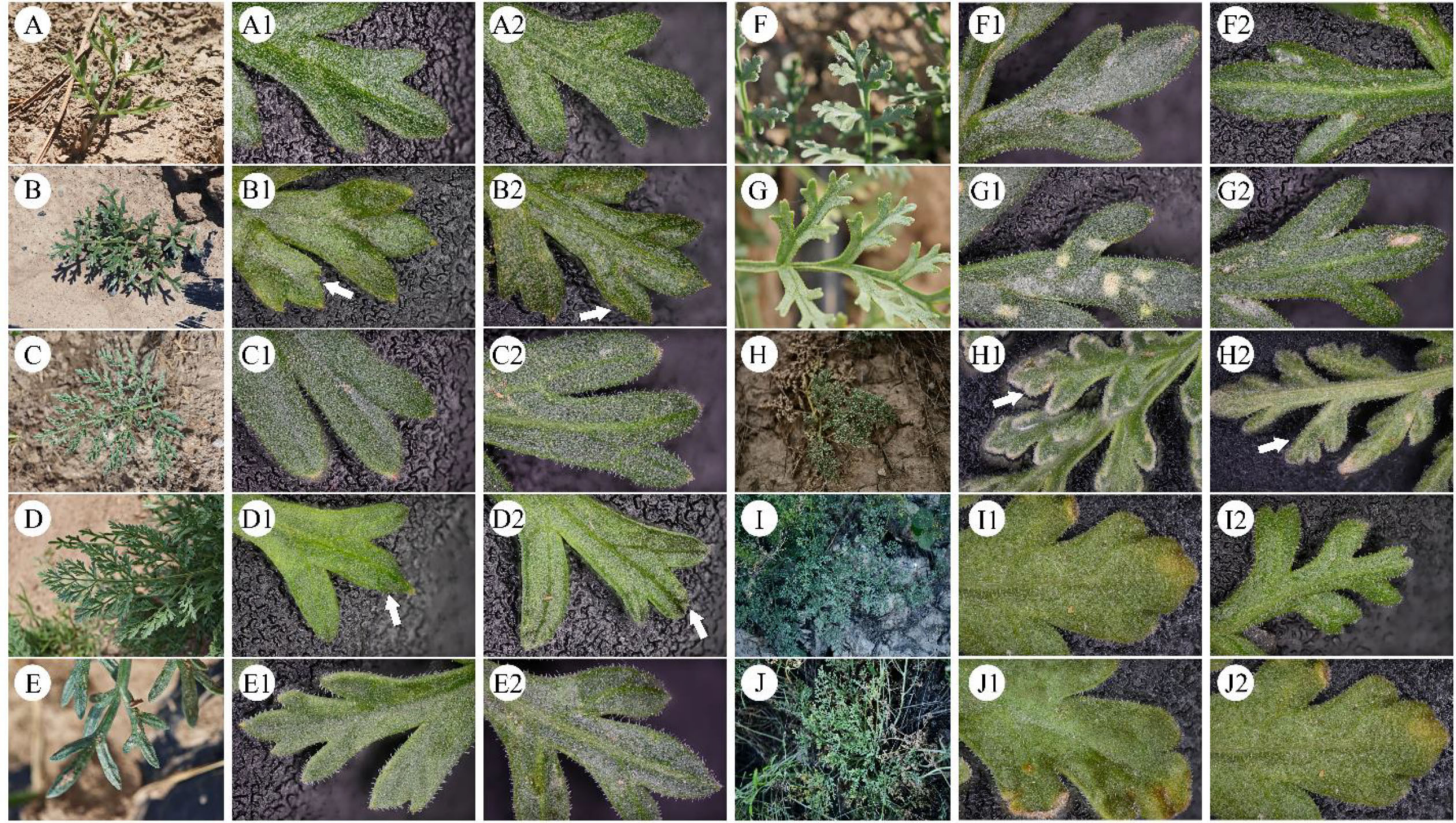
Leaf morphology of cultivated *Ferula* species. **(A)** YJH; **(B)** YNJDDG; **(C)** TJZTL; **(D)** TJZDG; **(E)** YNJDTL; **(F)** SHY; **(G)** ZJZ; **(H)** XDX; **(I)** FKXJ; **(J)** FKFK; **(A1–J1)** The adaxial surface of the leaf. **(A2–J2)** The abaxial surface of the leaf.

**Figure 3:**
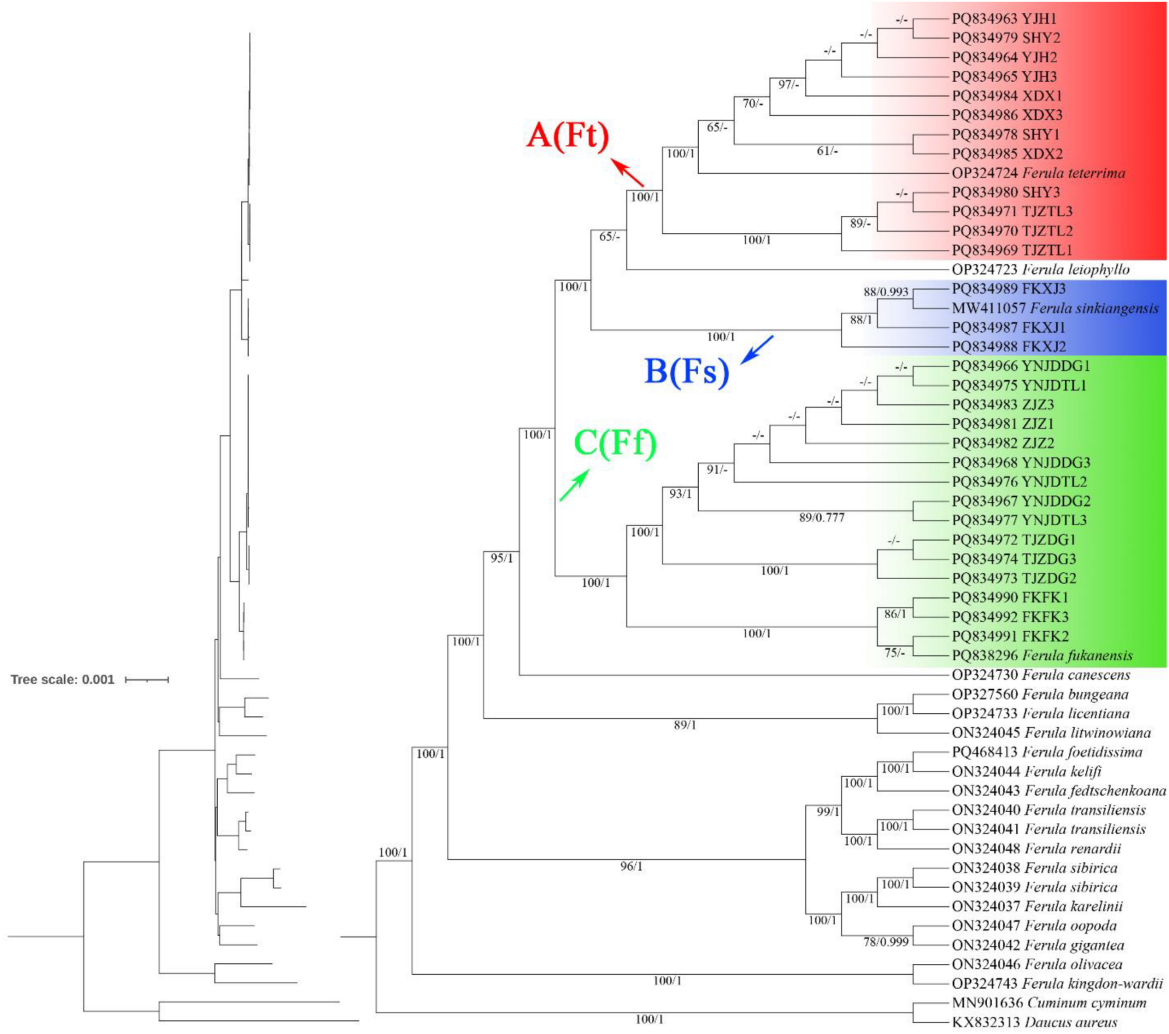
Phylogenetic relationships among cultivated *Ferula* species inferred from maximum likelihood (ML) and Bayesian inference (BI) analyses using complete chloroplast genome data. The topology includes a branch length scale (upper left) and a detailed phylogenetic tree. Branch support values are indicated as ML bootstrap support (BS)/Bayesian posterior probability (PP).

**Figure 4:**
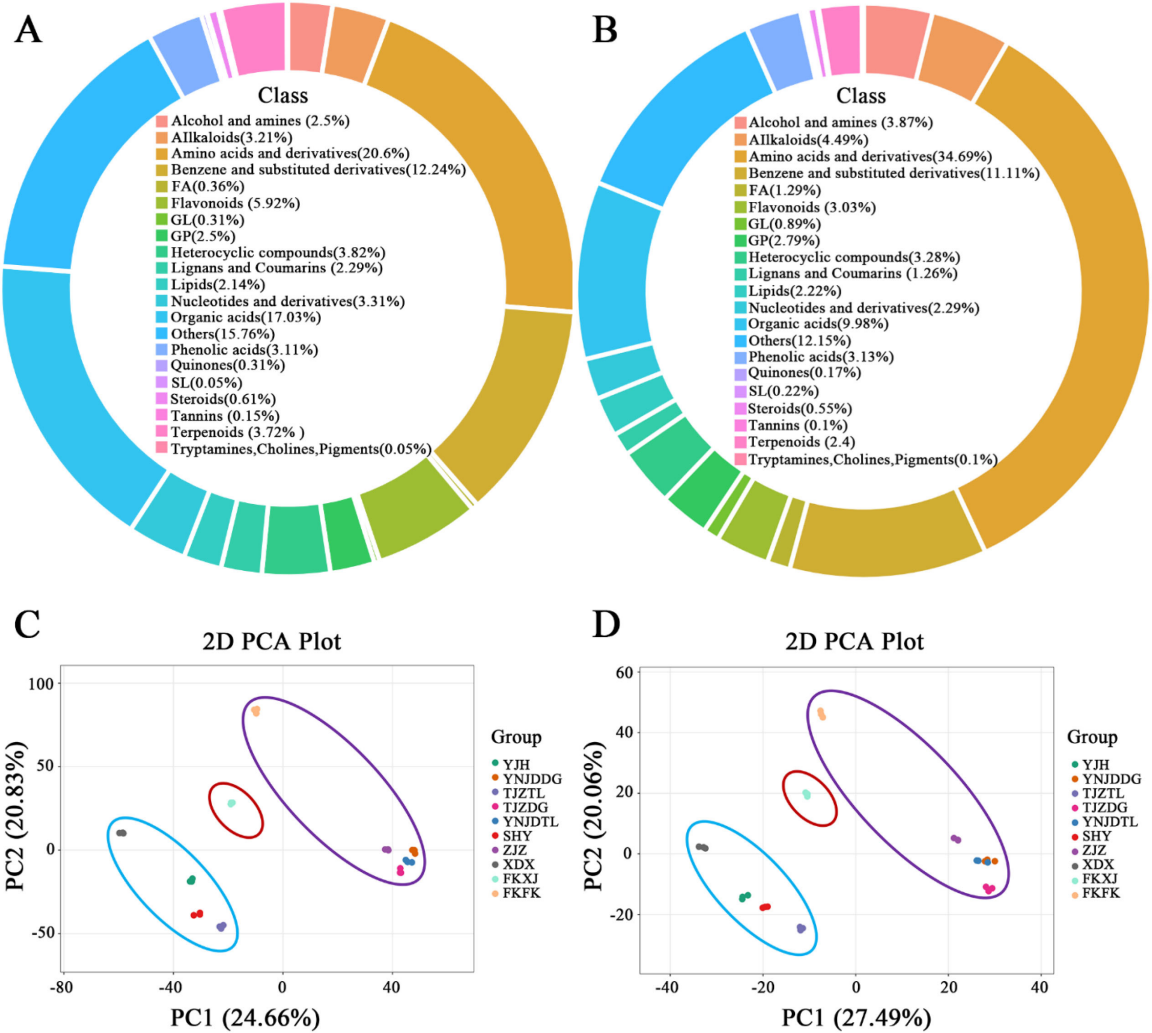
Metabolomic profiles and PCA of cultivated *Ferula* species based on UPLC-MS/MS data in **(A)** positive ion mode and **(B)** negative ion mode. **(C)** PCA scores plot in positive ion mode and **(D)** PCA scores plot in negative ion mode, illustrating species separation along PC1 and PC2. FA, Fatty Acids; GL, Glycerolipids; GP, Glycerophospholipids; SL, Sphingolipids.

**Figure 5:**
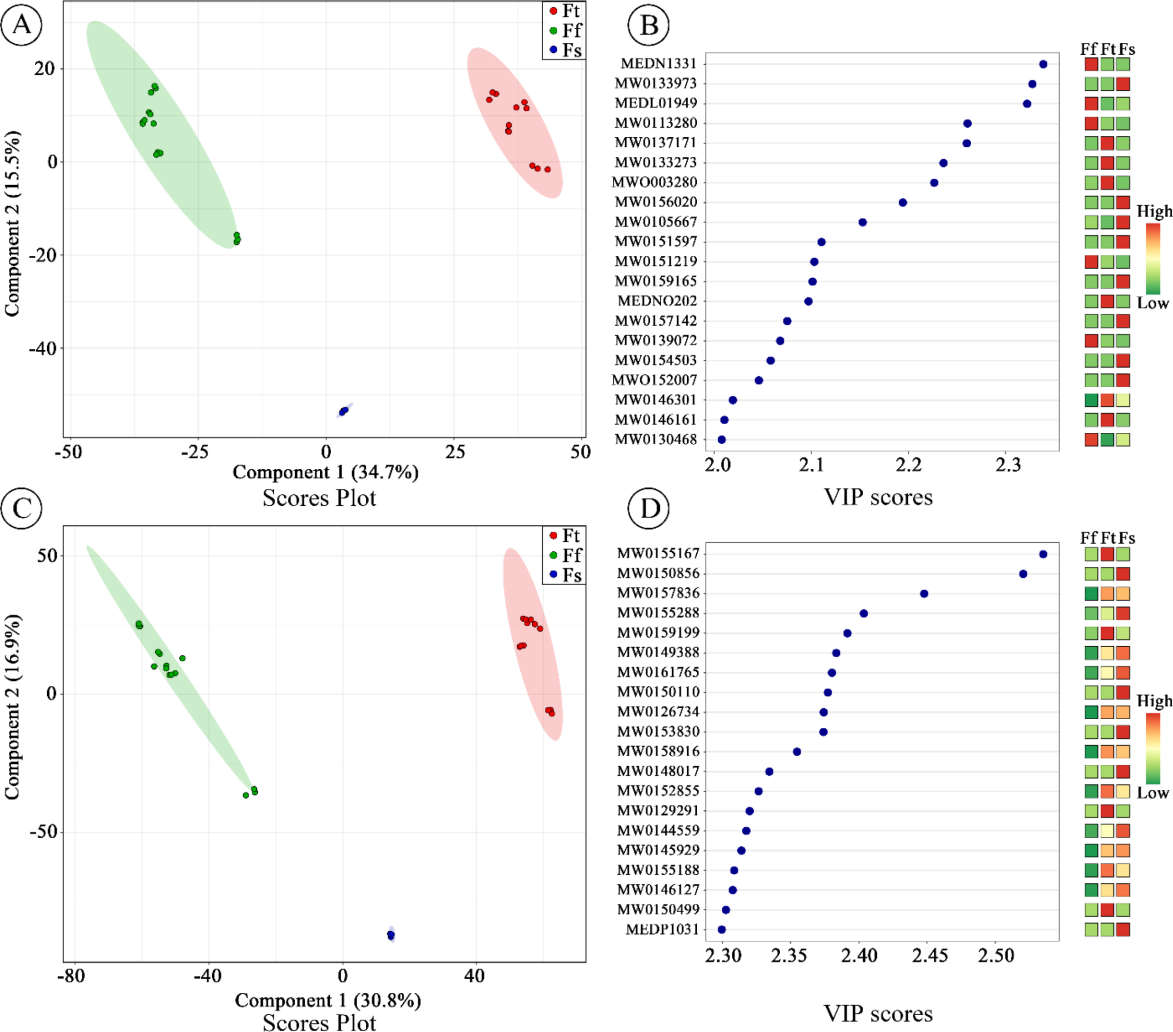
PLS-DA analysis of Ferula species based on UPLC-MS/MS metabolomic profiles in positive ion mode and negative ion mode. **(A)** PLS-DA score plot of positive ion mode for the first and second variables enlarged the discrimination between *Ferula* species, together with their respective 95% confidence regions. **(B)** The highest variable importance in projection (VIP) scores, the column on the left part indicates the metabolites, and coloured boxes on the right indicate the intensity level of the corresponding chemical feature in each group in positive ion modes. **(C)** PLS-DA score plot of negative ion mode for the first and second variables enlarged the discrimination between Ferula species, together with their respective 95% confidence regions. **(D)** The highest variable importance in projection (VIP) scores, the column on the left part indicates the metabolites, and coloured boxes on the right indicate the intensity level of the corresponding chemical feature in each group in negative ion modes. (*Ferula teterrima*: Ft, *Ferula fukanensis*: Ff, and *Ferula sinkiangensis*: Fs).

**Figure 6:**
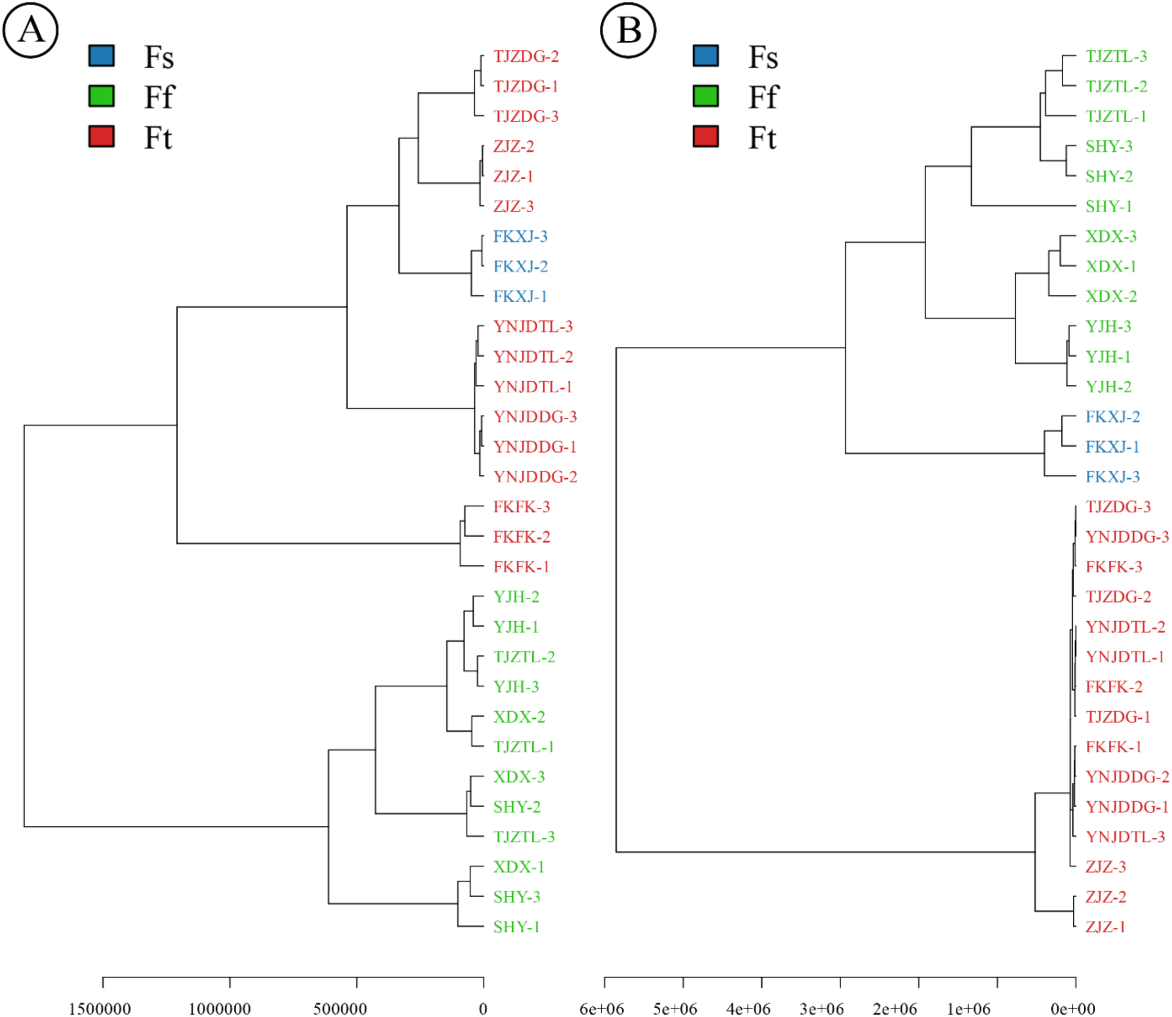
Chemotaxonomic classification of cultivated *Ferula* species based on the top twenty metabolites identified from PLS-DA loading plot scores (VIP > 1.0), integrating **(A)** Positive ion mode ESI+ and **(B)** negative ion mode ESI- mass spectrometry data.

## 3 Results and discussion

### 3.1 Morphological study of *Ferula* species

The morphological comparisons of *Ferula* species can’t reveal notable variations in leaf morphology differences across the examined samples (**Figure 2**). Images A–J represent the general habit and detailed leaf morphology of different specimens examined in this study. At the same time, subpanels (A1–J2) in (**Figure 2**) highlight adaxial and abaxial views of key morphological features including lobation, venation and surface pubescence. The collected sample leaflets are all trilobate and 2–3 times pinnately lobed. Through macro photography observation, it was found that the upper and lower surfaces of A, C, E, F, G, I, J have short, soft hairs, and the leaf margin and veins are very distinct. The leaf underside has significantly more hair than the upper surface. B and D only have short, soft hairs visible along the leaf margin (**Figure 2B**); H has dense long, soft hairs on both the upper and lower surfaces of the leaf, with the underside again having significantly more hair than the upper surface (**Figure 2**). Subsequently, key morphological features such as growth form, leaf arrangement and shape, venation, inflorescence structure, color and shape of flowers and fruits, were primarily used for preliminary identification of *Ferula* species as described in various taxonomic treatments of *Ferula* and Apiaceae (Korovin et al., 1951; Sheh et al., 2005). The traditional classification of *Ferula* species is mainly based on the morphological and anatomical characteristics (Korovin et al., 1951; Safina and Pimenov, 1983). However, in some cases, morphological differences between species are very small, while significant intraspecific variation can occur due to environmental influences, which significantly affects the species identification (Des Roches et al., 2018; Wang et al., 2022). For example, in this study, samples A, C, F, and H were identified as *F. teterrima* Kar. & Kir. based on our phylogenetic analysis (**Figure 3**). However, sample H exhibited distinct long, soft hairs likely influenced by its age (8 years) and its growth in a highly arid, undisturbed slope environment with minimal human interference. The phenotype of sample H may reflect morphological plasticity in response to local conditions, further reinforcing the need for molecular and metabolomic data for accurate identification. Furthermore, samples B, D, E, and G were all identified as *F. fukanensis* K.M.Shen based on phylogenetic analyses (**Figure 3**), but they could not be distinguished from one another based on their pubescence (hairiness) or leaf shape (**Figure 2**). Cultivated *Ferula* plants are typically harvested in batches, primarily for their young leaves, which makes it impossible to accurately determine the provenance of the germplasm just by examining the young leaves. Morphology serves as a preliminary criterion for plant identification (Kaplan, 2001), while molecular and metabolomic evidence can provide more reliable methods for accurately identifying complex plant species (CBOL Plant Working Group et al., 2009; Yang et al., 2013; Du Preez et al., 2025).

### 3.2 Phylogenetic analysis of *Ferula* species

In this study, based on complete plastome sequences, 53 sequences from *Ferula* were used to construct the ML and BI trees (**Figure 3**). The topologies of the ML and BI phylogenetic trees were consistent, and most nodes have strong support values (PP ≥ 0.997, BS ≥ 99%) therefore, only the ML bootstrap support (BS) and Bayesian posterior probability (PP) values indicated at each node (**Figure 3**). The ML phylogenetic tree illustrates the evolutionary relationship among *Ferula* species based on whole plastome genomic data. The cultivated *Ferula* cluster within three major clades: clade A (red), clade B (blue), and clade C (green) (**Figure 3**). The samples YJH, XDX, TJZTL, and SHY clustered together with *F. teterrima* to form clade A. Clade B consists of FKXJ (FKXJ1, FKXJ2, FKXJ3) with *F. sinkiangensis*, confirming their distinct lineage. The samples YNJDTL, YNJDDG, ZJZ, TJZDG, and FKFK, along with *F. fukanensis*, formed clade C, and the relationships of the three clades were well-resolved with strong support (BS = 100%, PP = 1) (**Figure 3**). Furthermore, the results of nuclear gene phylogenetic analysis based on ML and BI phylogenies showed that clade B (blue) exhibited a polyphyletic relationship, with lineages embedded within clade A (red). In contrast, clade C (green) retained a strong monophyletic relationship (**Supplementary Figures 7**, **8**).

Multiple accessions of the same species are labeled according to **Supplementary Table 1**. Chloroplast genomic data supported the identification of cultivated *Ferula* species into three clades corresponding to *Ferula teterrima* (A/Ft), *F. sinkiangensis* (B/Fs), and *F. fukanensis* (C/Ff) in the phylogenetic tree. Phylogenetic tree nodes marked with -/- indicates support less than 50).

Accurately delineating species boundaries is a crucial step in exploiting and conserving plant species (Guo et al., 2022; Zhao et al., 2025). *Ferula* is a medicinal plant, cultivated for the pharmaceutical industry and rural development; however, its taxonomic delimitation remains ambiguous. The monocarpic nature of *Ferula* significantly limits access to reproductive structures, which are critical for traditional taxonomic identification (Pimenov and Leonov, 1993; Downie et al., 2010). Accurate identification of *Ferula* requires complete, mature specimens including roots, stem bases, basal leaves, inflorescences, flowers, and ripe fruits. Relying solely on young basal leaves, particularly in early growth stages, is highly unreliable and often leads to misidentification (Panahi et al., 2018; Negrin et al., 2019). Furthermore, repeated harvesting of cultivated specimens for leaf material further reduces the availability of diagnostic morphological characters in market samples. These constraints render conventional morphological assessment unreliable for species delimitation in this genus. DNA barcoding enables direct comparison of genetic differences between species (Tan et al., 2020). Since its introduction by Canadian biologist Paul Hebert (Costa et al., 2007; Hebert et al., 2003; Liu et al., 2025a), DNA barcoding has advanced rapidly. For plants, commonly proposed plastid DNA barcodes include: *mat*K, *rbc*L, *trn*H-*psb*A, and ITS (Chase et al., 2005; Kress et al., 2005; Cowan et al., 2006; Xue et al., 2024). The first-generation plant DNA barcode, based on 2–3 plastid fragments and the ribosomal ITS sequence, has significantly advanced plant species identification, biodiversity research and evolutionary studies (Cowan et al., 2006; Dodsworth, 2015). Our nuclear gene phylogeny exhibits topological discordance with the plastome-based phylogeny, particularly with *F. sinkiangensis* being nested within the *F. teterrima* clade (**Supplementary Figures 7, 8**). Two plausible explanations may account for this discordance. The first is taxonomic: as noted in the Flora of Xinjiangensis, when *F. sinkiangensis* was originally described, some authors have subsequently treated *F. sinkiangensis* as synonymous with *F. teterrima.* The Flora emphasizes that *F. sinkiangensis* can be distinguished from *F. teterrima* by its multiple vittae in the fruit, a character that has led most recent taxonomic treatments to maintain them as separate species. However, our phylogenetic data alone cannot definitively resolve this long-standing taxonomic ambiguity (Shen, 2011). Second, the chloroplast genome is maternally inherited and may exhibit evolutionary asynchrony with the nuclear genome. Potential biological factors leading to such discordance include incomplete lineage sorting (ILS) and gene introgression (Zhao et al., 2023; Liu et al., 2025b). Given that the sampled *Ferula* individuals had not flowered or exchanged genes prior to collection, contemporary gene flow can be largely excluded, making ILS a more plausible explanation than recent hybridization or chloroplast capture. ILS occurs when ancestral polymorphisms persist through successive speciation events and are randomly sorted into descendant lineages, leading to gene trees that deviate from the species tree. This phenomenon is particularly pronounced in lineages that underwent rapid radiation, such as *Ferula*, where coalescence times may be long relative to speciation intervals (Kurzyna-Młynik et al., 2008; Panahi et al., 2015, 2018). Additionally, the nuclear ITS/ETS fragments analyzed were relatively short and contained limited phylogenetically informative sites. This resulted in low nodal support across much of the *Ferula* phylogeny (**Supplementary Figures 7**, **8**), particularly within taxonomically complex clades, further obscuring relationships among closely related species. The limitations of first-generation barcodes in resolving complex groups like *Ferula* highlight the need for more comprehensive approaches. Therefore, for authenticating cultivated *Ferula* germplasm, we utilized whole plastome genomic data to provide a more robust and reproducible framework (**Figure 3**). Using the plastome as a super-barcode offers a powerful solution, enabling more effective species identification within such challenging taxa (Liu et al., 2021; Lv et al., 2023; Dewar et al., 2025), even as the full evolutionary history remains partially unresolved.

### 3.3 Metabolic profiles of *Ferula* species via UPLC-MS/MS

Metabolomics is a powerful tool for identifying and characterizing medicinal plants through the analysis of their chemical profiles. Therefore, when integrated with phylogenetic data, metabolomic profiling provides powerful complementary evidence for species identification and chemotaxonomic classification. It helps differentiate plant species, validate herbal products, and explore their therapeutic potential by identifying key metabolites. Techniques like LC-MS, GC-MS, and metabolomics analyses offer insights into plant variability, aiding in the authentication and quality control of herbal medicines (Wallace et al., 2020; Wang et al., 2023e; Zhang et al., 2023). The UPLC-MS/MS, an untargeted metabolomics approach, renowned for its superior sensitivity and separation efficiency, was employed to profile metabolites (Lommen et al., 2007). Standardized extraction and dilution protocols were used to ensure unbiased analysis. Data processing steps like background subtraction and peak alignment were followed to improve the accuracy and reliability of metabolite identification. Reproducibility was confirmed by overlaying extracted ion chromatogram (EIC) and total ion chromatograms (TICs) analysis in UPLC-MS/MS in negative mode (**Supplementary Figures 9**, **10**) and positive mode, respectively (**Supplementary Figures 11**, **12**), which showed consistent retention times (RT) and peak intensities. The metabolic analysis was performed on 30 *Ferula* samples from 10 cultivated locations, which were then divided into 10 clusters for additional comparison. In total, 7,169 metabolites (**Supplementary Table 2**) were detected across both ionization modes, with 5,600 metabolites identified in positive ion mode and 1,569 metabolites in negative ion mode through an untargeted metabolomic approach using liquid chromatography-mass spectrometry (LC-MS). Among these, the metabolites classes in positive and negative ion modes, respectively, included Alcohol and amines (2.5%, 3.87%), Alkaloids (3.21%, 4.49%), Amino acids and derivatives (20.6%, 34.69%), Flavonoids (5.92%, 3.03%), Organic acids (17.03%, 9.98%), Phenolic acids (3.11%, 3.13%), Terpenoids (3.72%, 2.4%), etc. (**Figures 4A, B**). The accumulation of secondary metabolites varies across plant species (Wang et al., 2016), reflecting their role in plant fitness and adaptation to environmental conditions (Hoffmann et al., 2017). These specialized metabolites exhibit species- or group-specific profiles, yet their biosynthesis can be influenced by environmental factors such as climate (temperature, light, and water) and soil properties (Negrin et al., 2019). Similarly, dynamic nature of metabolite composition was observed in the metabolic profiles across both ionization modes of *Ferula* species (**Figures 4A, B**). The metabolomic differences identified among the *Ferula* species likely primarily reflect inherent species-specific biochemical pathways with minimal confounding effects from phenotypic plasticity induced by environmental or developmental variation. This interpretation is supported by our controlled sampling design, wherein all leaf tissues were collected from plants at a uniform developmental stage and grown under the same agricultural conditions. Consequently, the distinct metabolic profiles we report can be more confidently attributed to genetic divergence among the studied species. However, we acknowledge a limitation of this study: while our sampling strategy controlled for macro-environmental factors, it cannot entirely rule out the influence of micro-environmental heterogeneity (e.g., localized differences in soil rhizosphere or light exposure) or unaccounted-for epigenetic modifications that could also contribute to the chemotypic variation. Future studies employing common garden experiments or controlled environment studies would be necessary to definitively disentangle the genetic and environmental components of the observed metabolomic diversity. These results underscore the capacity of the advanced UPLC-MS/MS method to capture extensive chemical information from studied samples, which will be valuable for classification, and understanding the metabolic diversity of *Ferula* species. The detection of a higher number of metabolites in the positive ionization mode compared to the negative mode suggests a preferential ionization of certain compounds. This trend is consistent with findings from other plant metabolomics studies, where compounds such as Alcohol, amines, and amino acids derivatives ionize more efficiently in positive mode, while other metabolite classes, such as certain organic acids and flavonoids, may produce stronger signals in negative mode (Hu et al., 2020; Zhang et al., 2023).

#### 3.3.1 PCA and PLS-DA

Multivariate analysis, including PCA and PLS-DA was performed using UPLC-MS/MS data obtained through XCMS software to assess the heterogeneity of metabolites among *Ferula* accessions and to classify the studied cultivated species of *Ferula*. The PCA (**Figure 4**) analysis of 30 *Ferula* samples revealed a clear separation into three distinct clades, which were assigned to *F. fukanensis* (YNJDDG, TJZDG, YNJDTL, ZJZ and FKFK), *F. sinkiangensis* (FKXJ), and *F. teterrima* (YJH, TJZTL, SHY and XDX) based on the phylogenetic tree (**Figure 3**). This clustering closely aligned with the clades identified in the phylogenetic analysis based on plastome sequences, highlighting the consistency between metabolomics and genetic data results. PCA, an unsupervised pattern recognition method, is commonly employed to reduce the dimensionality of complex numerical datasets while preserving essential variance (Farag et al., 2015; Fayek et al., 2021). It is widely used for the classification, quality assessment, and discrimination of herbal medicinal plants. Recent studies have highlighted its effectiveness in distinguishing botanical raw materials like *Panax notoginseng* and authenticating plant materials from various geographical regions using metabolomics data (Zhu et al., 2014; Zhang et al., 2023). The PCA (**Figure 4**), based on the UPLC-MS/MS dataset, illustrates the clear discrimination of species, as evidenced by the tight clustering of individual replicates within each sample. In the PCA score plot for positive ion mode (**Figure 4C**), the first two principal components (PC1 and PC2) together account for 45.49% of the total variance. PC1 accounted for 24.66% of the variance, while PC2 contributed 20.83%. This aligns with the studies (Souard et al., 2018) that multivariate statistical analysis involving multiple groups with multiple detection objects can lead to a lower variance explained by the forward PCs. The *Ferula* accessions were categorized into three distinct clades, each reflecting unique metabolic profiles and highlighting differences among the studied species. In negative ion mode (**Figure 4D**), the first two principal components (PCs) accounted for 47.55% of the total variation among the samples in the PCA. The PCA plot showed that species including YNJDDG, TJZDG, YNJDTL, and ZJZ clustered closely together, suggesting a high level of metabolic homogeneity. This pattern aligns with the phylogenetic relationships constructed in this study (**Figure 3**). Despite this homogeneity, FKFK showed a distinct separation in the PCA (**Figures 4C, D**). However, phylogenetic analysis confirmed that FKFK belongs to the same species, identified as *F. fukanensis*. In contrast, FKXJ exhibited notable metabolic heterogeneity and showed distinct separation from the other groups (**Figure 3**), aligning with its phylogenetic position (**Figure 3**) and supporting its classification as a separate species, *F. sinkiangensis*. Additionally, species including YJH, TJZTL, SHY, and XDX demonstrated close clustering in the PCA plot, indicating minimal variation in their metabolite profiles. These results are consistent with phylogenetic analyses, which identified these species as belonging to *F. teterrima*. The results of PCA support the inferred relationships observed for the *Ferula* species based on the phylogenetic relationship (**Figure 3**). Similarly, employing UPLC-MS metabolomics has been effective in discriminating the diversity of metabolites at the intrageneric level among *Vicia* species, revealing potential chemotaxonomic markers for their discrimination (Fayek et al., 2021). Negrin et al. studied the metabolomics of Holly (*Ilex)* species and related taxa in the Aquifoliaceae family, with results demonstrating that *Ilex* species can be differentiated through metabolite profiling (Negrin et al., 2019).

Plant secondary metabolites frequently exhibit taxon-specific chemical structures and biosynthetic pathways, strengthening their utility in taxonomic classification (Endara et al., 2018; Shen et al., 2024). The PLS-DA score plots (**Figure 5**), a supervised pattern recognition method, was carried out to distinguish among different cultivated *Ferula* species. PLS-DA reveal distinct clustering patterns among taxonomic groups (*Ferula teterrima*: Ft, *Ferula fukanensis*: Ff, and *Ferula sinkiangensis*: Fs), underscoring significant metabolic differentiation driven by taxonomic affiliation through phylogenetic analysis based on plastome data (**Figure 3**). PLS-DA displays separation with two components of 34.7% and 15.5% variance, isolating XJ from Ft/Ff (**Figure 5A**) in positive ion mode, while in negative ion mode with the first two components of 30.8% and 16.9% in score plot, revealed separation of all groups. (**Figure 5C**). These divergent clustering patterns suggest taxonomic-specific metabolic signatures, consistent with prior observations that secondary metabolite profiles correlate strongly with phylogenetic relationships (**Figure 3**). Furthermore, to decipher the molecular basis of observed separations in our dataset, we prioritized variables using variable importance in projection (VIP) scores derived from PLS-DA. VIP values quantify the relative contribution of individual metabolites to class discrimination, with higher scores indicating stronger discriminatory power. In this study, the top 20 metabolites demonstrated exceptional discriminative capacity, achieving VIP thresholds of >2.5 (positive ionization mode) and >2.0 (negative mode) (**Figures 5B, D**). The compound annotated as Phe-Gln-Ser was among the top metabolites contributing to the separation of Ft from other species in this dataset (e.g., MW0155167), 1-Phenylethanol for Fs samples (e.g., MEDP1031) in positive mode, and 4-Methoxyphenyllactic acid for Ff samples in negative mode (e.g., MEDN1331). These results suggest that the metabolic patterns captured by PLS-DA could serve as a reference for distinguishing among Fs, Ff, and Ft in a controlled, exploratory context. However, as no independent test set was used, these models require validation with external samples before any predictive application. Nonetheless, the identification of species-associated metabolites (e.g., Phe-Gln-Ser for Ft, 1-Phenylethanol for Fs) provides strong chemotaxonomic markers that support the genetic distinctions revealed by plastome phylogenomic.

Furthermore, a dendrogram of metabolomic profiles under positive (A) and negative (B) ion modes (**Figure 6**) was consistent with the PLS-DA findings, confirming species-specific chemotaxonomic separation among Fs, Ff, and Ft samples. In positive ion mode, Fs forms a separate clade within Ft, while Ff shows clear separation from other samples, while Fs and Ft clustered separately with minimal overlap. In negative ion mode, separation remained evident, though Fs and Ff displayed closer proximity, reflecting partial overlap in metabolite composition. The clustering analyses support the robustness of PLS-DA models, demonstrating that cultivated *Ferula* species possess distinct metabolomic fingerprints that align with taxonomic boundaries consistent with previous chemotaxonomic studies (Chilaka et al., 2025).

Beyond serving as a supportive line of evidence for taxonomy, these metabolomic distinctions have direct medicinal and authentication relevance, markers that segregate species correspond to compound classes (sesquiterpene coumarins, sulfur-containing resins) that underpin many reported bioactivities of *Ferula* (antimicrobial, anti-inflammatory, and enzyme-modulating effects) (Jiang et al., 2023; Wariss et al., 2026), so species-level misidentification could meaningfully alter pharmacological profiles of commercial products. The integration of plastome data with untargeted metabolomics provides a more robust framework for species identification and characterization in cultivated *Ferula*. Chloroplast genomes offer robust genetic resolution, while metabolomic profiles capture the biochemical phenotype. This dual approach enables the correlation of genotype with chemotype, facilitates the discovery of diagnostic markers, and supports the selection of high-value chemotypes. Crucially, it demonstrates how metabolic evidence can corroborate and add a functional dimension to phylogenetic hypotheses. Future research should focus on quantitative targeted assays, MS/MS-based metabolite annotation, and dense geographic and phenological sampling in order to put this into practice. This will lay the groundwork for standardized authentication and quality control in pharmaceutical and industrial applications.

#### 3.3.3 Hierarchical cluster analysis (HCA) of *Ferula* species

The HCA (**Supplementary Figure 13**) illustrates the metabolite profiles of 30 samples across ten groups, revealing distinct patterns of metabolic variation. To further visualize the chemical differences among the *Ferula* species, a heat map was generated based on metabolic analysis of UPLC-MS/SM in the negative ion mode, representing the metabolic variation across the 30 samples (**Supplementary Figure 13**). This approach highlights the species-specific metabolic signatures and provides a comprehensive view of the chemical diversity within the *Ferula* species. The Z-scores of metabolites were used to normalize and visualize the intensity data, with warmer colors (red/orange) indicating higher metabolite levels and cooler colors (green) representing lower levels. The samples clustered both by rows (metabolites) and columns (sample groups), reflecting similarities and differences in metabolic composition across groups.

HCA is an exploratory analysis that classifies variables by clustering samples with similar features, and separating samples with different features (Cheng et al., 2020; Shen et al., 2024). The HCA (**Supplementary Figure 13**) reveals three major clusters, highlighting distinct patterns of metabolic variation among the samples. The species TJZDG, ZJZ, YNJDDG, and YNJDTL formed a distinct clade, indicating high metabolic homogeneity and supporting their identification as *F. fukanensis*. This clustering pattern aligns with their proximity to the PCA plot (**Figure 4**) and is consistent with the phylogenetic relationships constructed in this study (**Figure 3**). In contrast, FKFK and FKXJ with the other four species formed two distinct clusters, highlighting the unique metabolic profile of these species. The HCA further identified a sister clade consisting of FKXJ, YJH, TJZTL, SHY, and XDX, indicating metabolic homogeneity within this subgroup. Notably, FKXJ is a sister species within this clade, revealing its distinct metabolic profile. This clustering pattern supports the recognition of these samples as distinct species identified as *F. teterrima* (YJH, TJZTL, SHY, and XDX) *and F. sinkiangensis* (FKXJ), respectively.

These findings support the coherence between the metabolomic profiles and the PCA and PLS-DA results, emphasizing the distinct chemical signatures and evolutionary relationships among the *Ferula* species. The structure of secondary metabolites and their biosynthetic pathways are often specific and restricted to taxonomically related organisms, making them useful for classification (Endara et al., 2018; García-Pérez et al., 2024). Phytochemical profiles were used to assess the Chemotaxonomic relationship of *Ferula* species reflecting underlying genetic morphology and its interactions with the environmental factors (Zhang et al., 2023; He et al., 2025). Metabolomic fingerprinting contributes to the classification of many crop plant species including date palm fruit (Farag et al., 2014) and *Vicia* species (Fayek et al., 2021). For instance, Metabolomics, combined with multivariate analyses, has proven effective in discriminating between *Camellia sinensis* (Ku et al., 2010), *Ilex* (Negrin et al., 2019) and blueberry samples (Ma et al., 2013).

## 4 Conclusion

Plant derived drugs have long faced challenges due to limited natural resources. Currently, artificial cultivation is the most practical approach to ensure a reliable supply of raw materials for natural medicines. However, challenges remain for multi-source medicinal materials like *Ferula*, used for culinary and medicinal purposes. Traditional morphological methods often fall short, especially when only vegetative parts are available. This is the first study to integrate morphological, genetic, and metabolic fingerprinting using a metabolome-based approach (UPLC-MS/MS) unified with chemometric tools such as HCA, PCA, and PLS-DA, to systematically delineate cultivated *Ferula* species in Xinjiang. Phylogenomic analysis based on complete plastome sequences clearly classified the studied accessions into three distinct clades: *F. sinkiangensis*, *F. fukanensis*, and *F. teterrima*. Untargeted UPLC-MS/MS metabolomic profiling further validated this classification, revealing significant interspecific differences in metabolite composition, with clustering patterns that strongly aligned with phylogenetic relationships. Our findings demonstrate that integrating phylogenomics with complementary metabolomic profiling provides a robust framework for species discrimination in cultivated *Ferula*, overcoming the limitations of traditional identification methods. Specifically, the metabolomic data offer chemotaxonomic support for the phylogenetic distinctions, aiding in species differentiation. The established plastome reference dataset also serves as a valuable resource for future germplasm monitoring and trade tracking. Collectively, this study establishes an integrated chemotaxonomic foundation for the standardized authentication and quality control of *Ferula* species and offers a replicable strategy for the classification and sustainable utilization of other medicinal plants.

## Data Availability

The datasets presented in this study can be found in online repositories. The names of the repository/repositories and accession number(s) can be found in the article/**Supplementary Material**.
