# Supplementary material for: Phylogenomic and metabolomic approaches to provide insight for species delimitation of cultivated Ferula species in Xinjiang, China
Source: Front Plant Sci. 2026 Apr 27;17:1754844. doi: 10.3389/fpls.2026.1754844 (PMC13158219; doi:10.3389/fpls.2026.1754844)
Supplement: Supplementary file 1 [file DataSheet1.docx]

**Supporting Information**

**Phylogenomics and metabolomics for species delimitation of cultivated *Ferula* species in Xinjiang, China**

Hafiz Muhammad Wariss^1,2,3,4,^**^†^**, Lei Yang^1,3,5,6,^**^†^**, Jinxin Gu^1,2,6^, Danhui Liu^1,2,3,5^, Wenjun Li^1,2,3,5,6*^

^1^State Key Laboratory of Ecological Safety and Sustainable Development in Arid Lands, Xinjiang Institute of Ecology and Geography, Chinese Academy of Sciences, Urumqi, China

^2^Xinjiang Key Laboratory of Biodiversity Conservation and Application in Arid Lands, Xinjiang Institute of Ecology and Geography, Chinese Academy of Sciences, Urumqi, China

^3^China-Tajikistan Belt and Road Joint Laboratory on Biodiversity Conservation and Sustainable Use, Xinjiang Institute of Ecology and Geography, Chinese Academy of Sciences, Urumqi, China

^4^Department of Botany, University of Sargodha, Sargodha, Pakistan

^5^ The Specimen Museum of Xinjiang Institute of Ecology and Geography, Chinese Academy of Sciences, Urumqi, China

^6^College of Resources and Environment, University of Chinese Academy of Sciences, Beijing, China

**^†^**Equally contributed.

*Corresponding author:

E-mail: [liwenjunao@ms.xjb.ac.cn](mailto:liwenjunao@ms.xjb.ac.cn) (Wenjun Li)

**Supplementary Figure 1.** PCA score plot (positive ion mode) showing the distribution of QC samples.

**
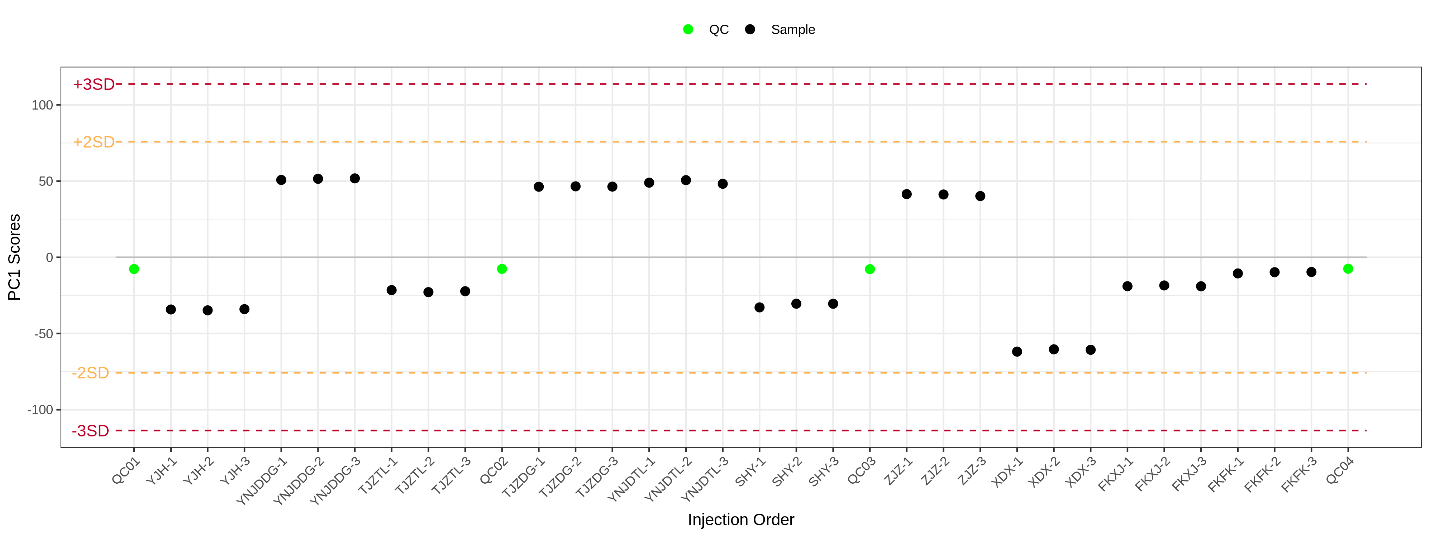
**

**Supplementary Figure 2.** PCA score plot (negative ion mode) showing the distribution of QC samples.

**
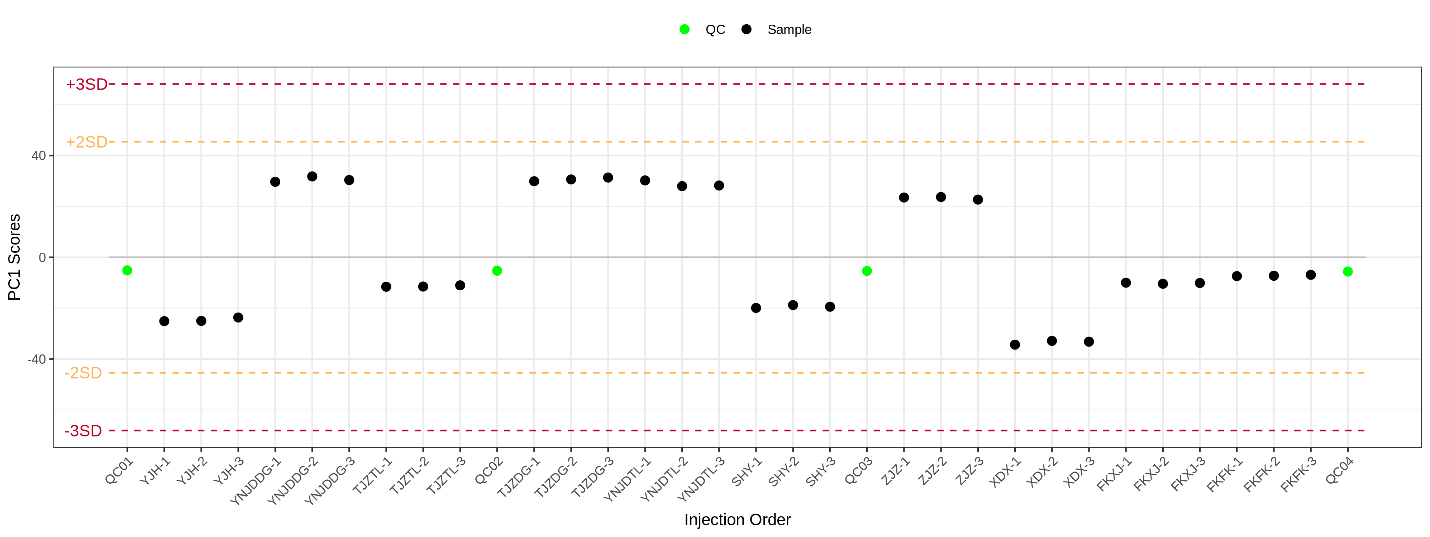
**

**Supplementary Figure 3.** PLS-DA model performance metrics in positive mode.


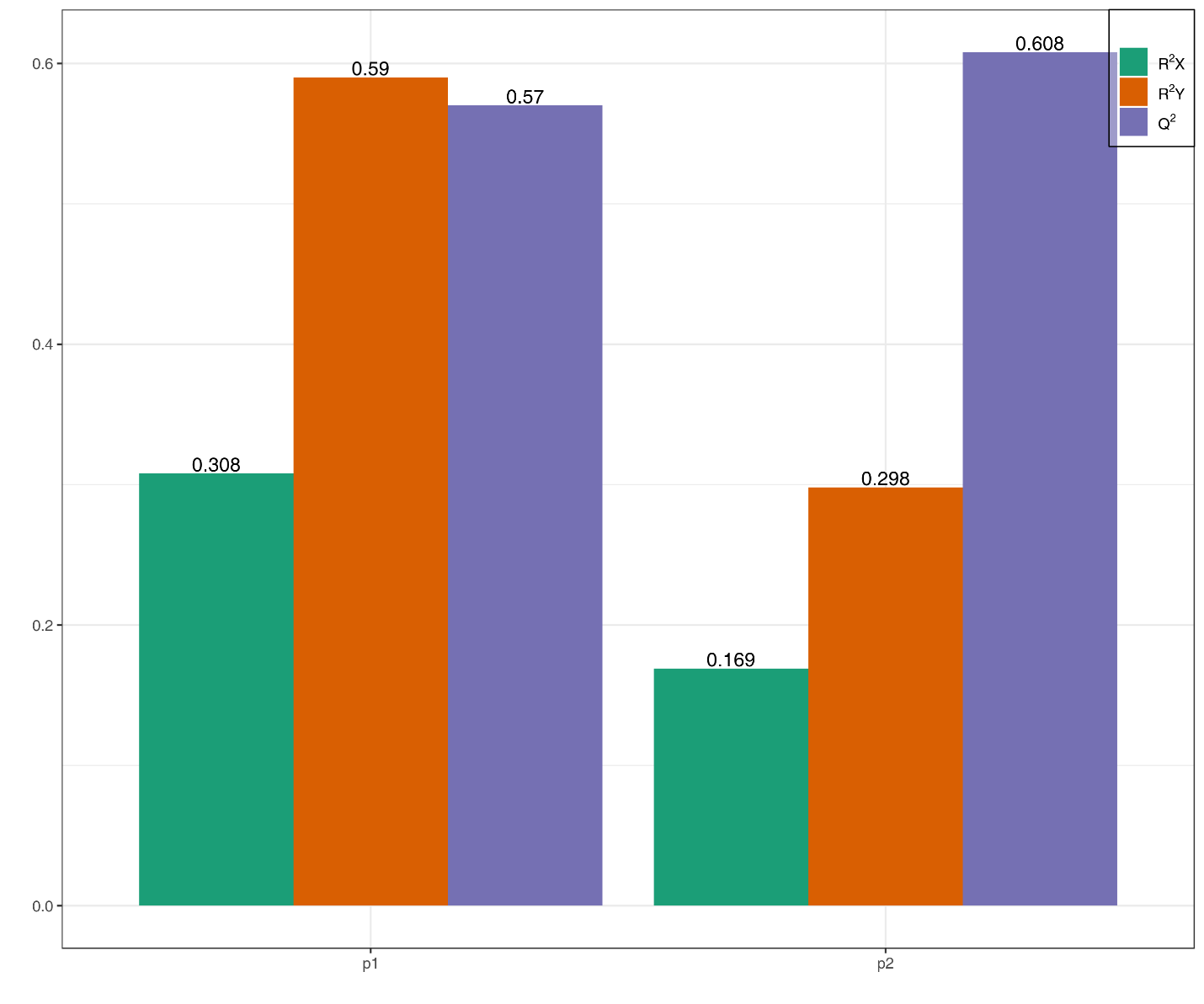


**Supplementary Figure 4.** Permutation test in positive mode.

**
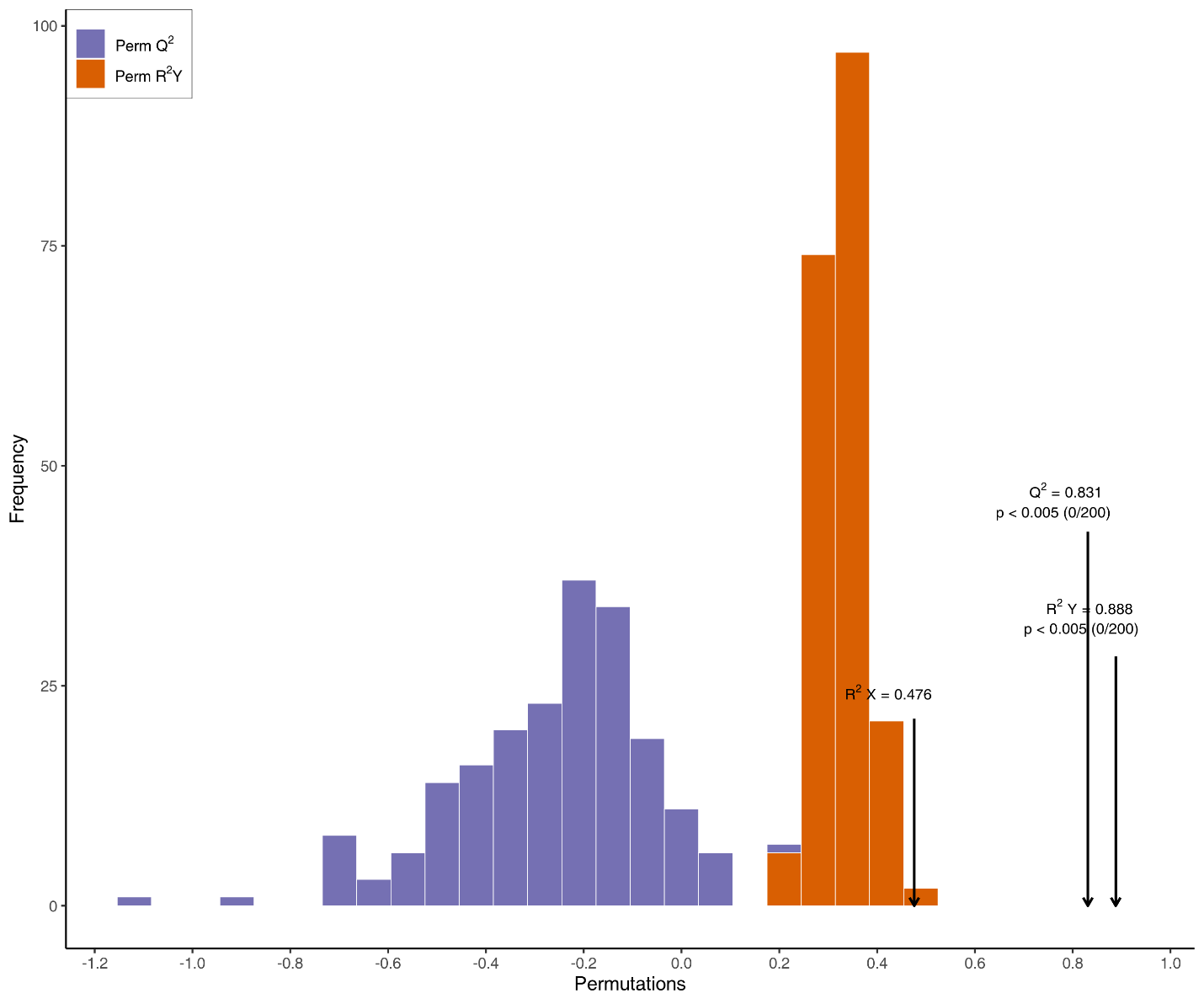
**

**Supplementary Figure 5.** PLS-DA model performance metrics in negative mode. **
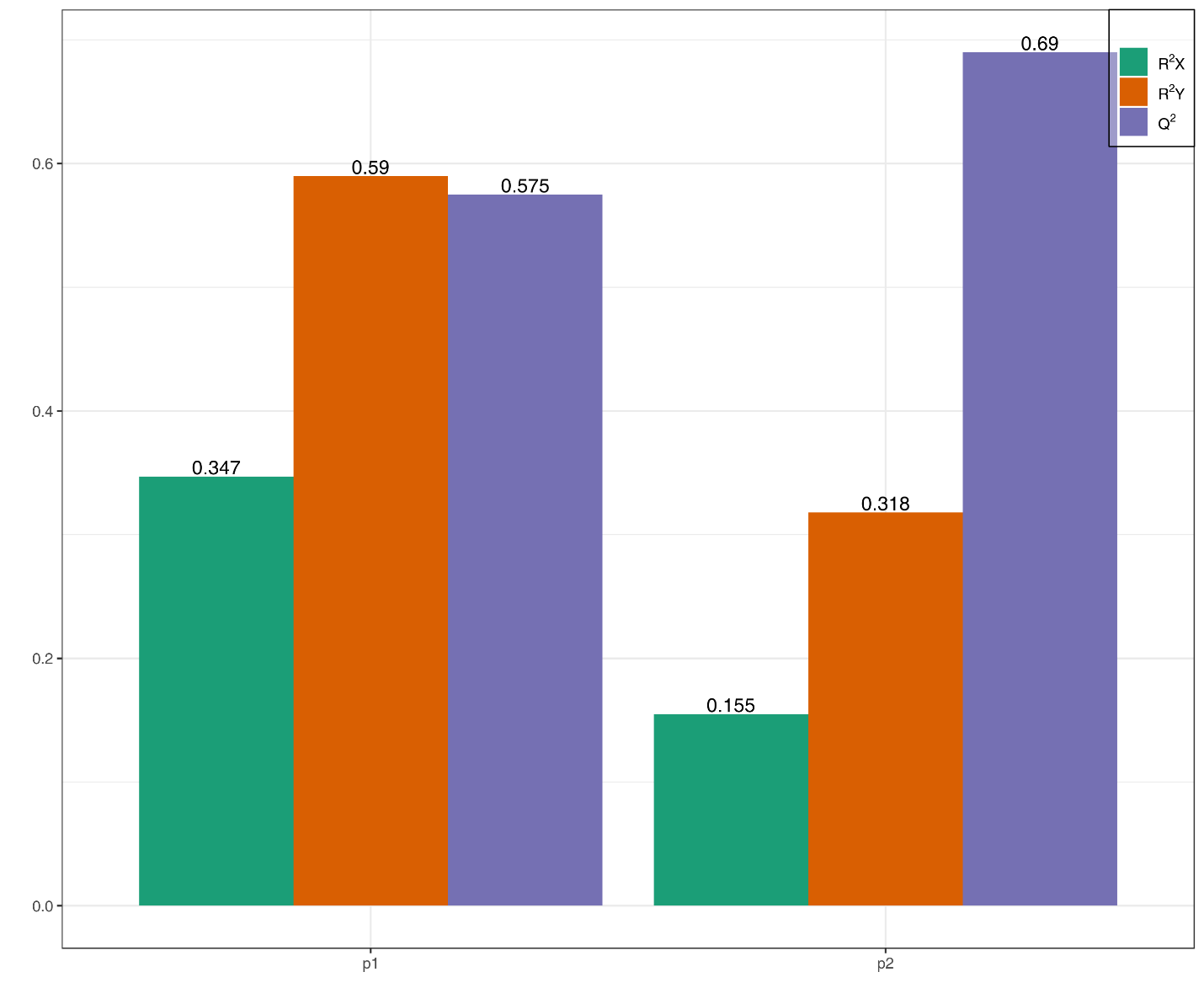
**

**Supplementary Figure 6.** Permutation test in negative mode.

**
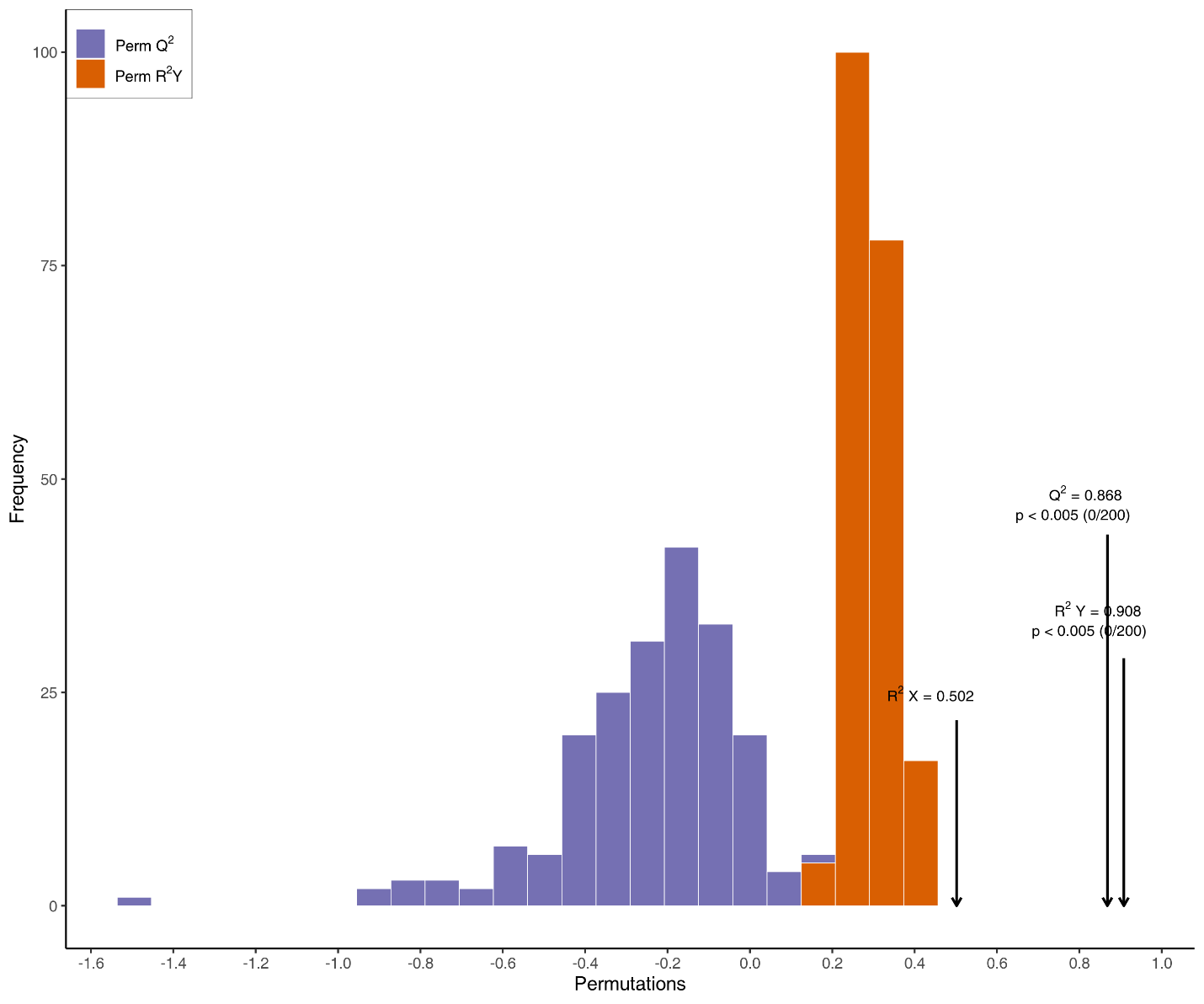
**

**Supplementary Figure 7.** Phylogeny of cultivated *Ferula* inferred by maximum likelihood (ML) based on the whole chloroplast dataset.


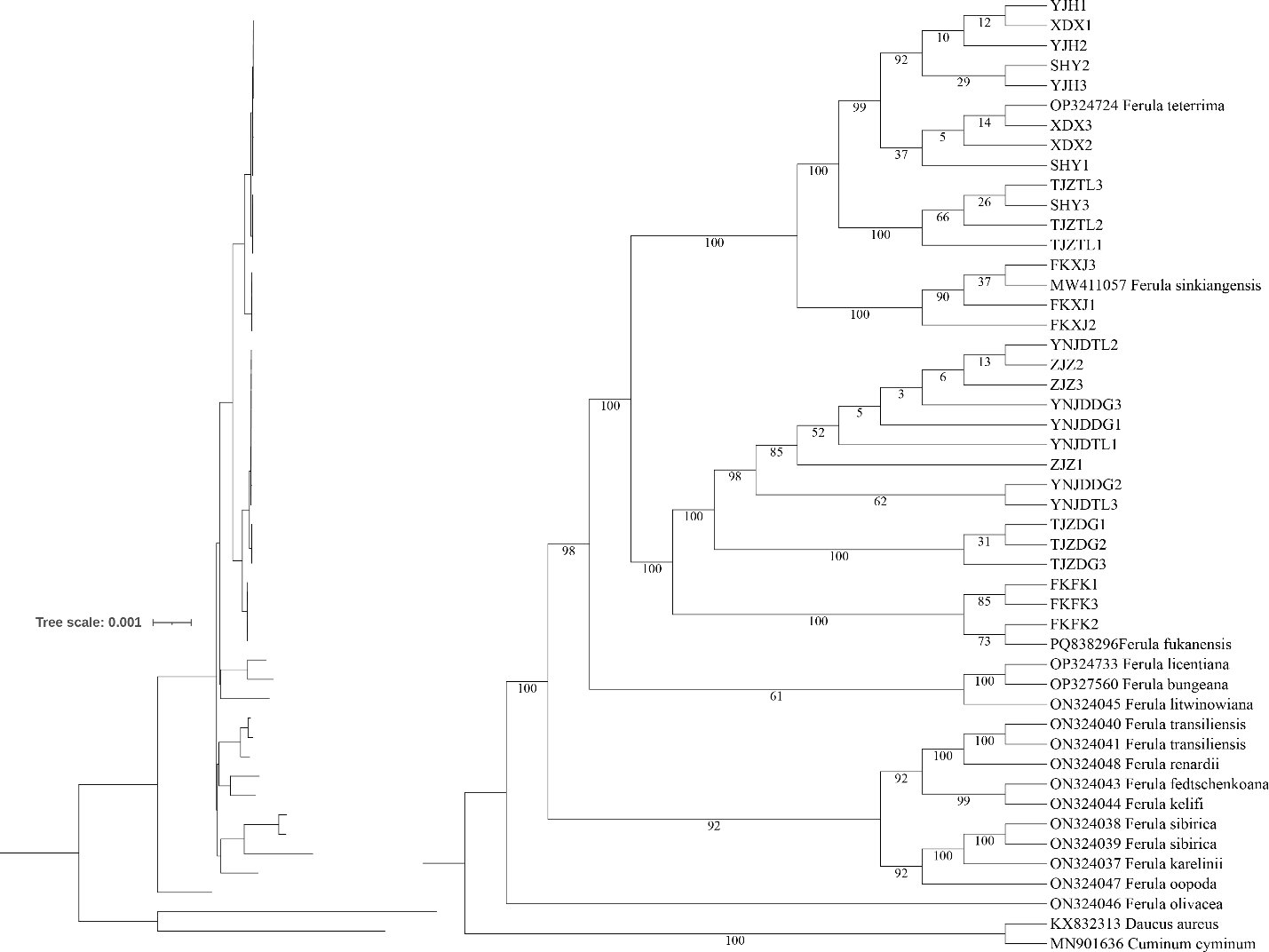


**Supplementary Figure 8.** Phylogeny of cultivated Ferula inferred by Bayesian inference (BI) based on the whole chloroplast dataset.


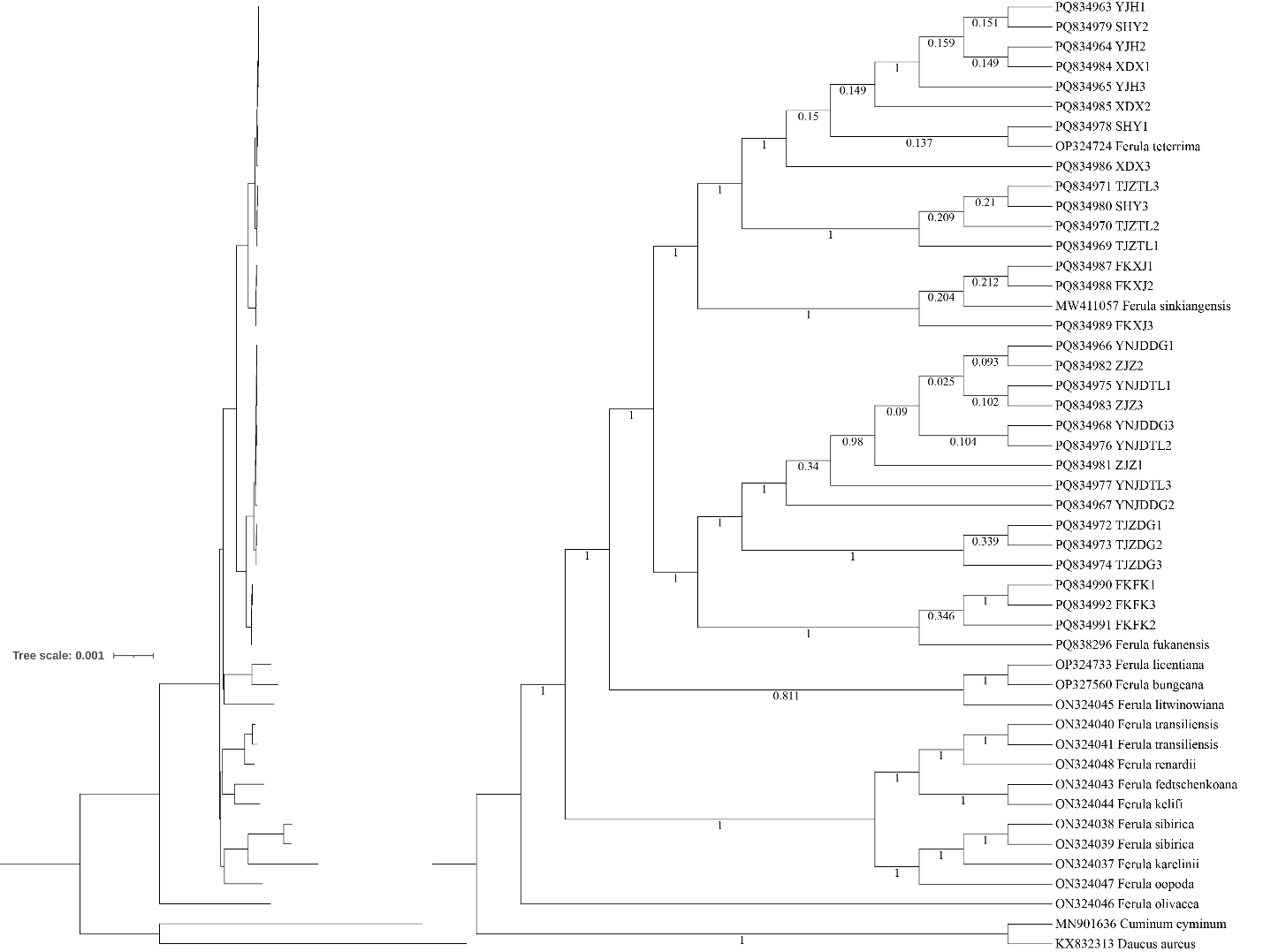


**Supplementary Figure 9.** Extracted Ion Chromatogram (EIC) of UPLC-MS/MS analysis in negative ion mode, showing the retention times and peak intensities of targeted ions in the sample.


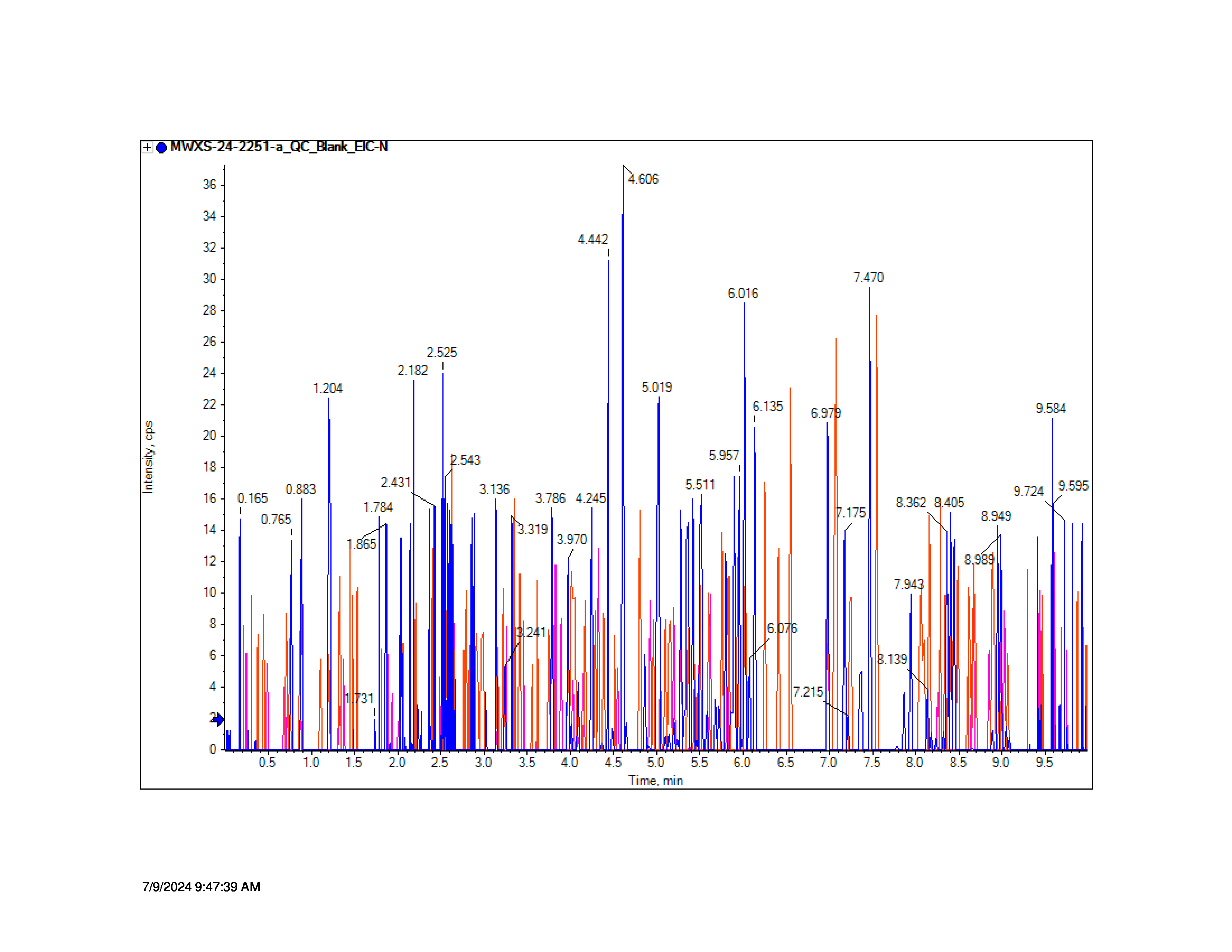


**Supplementary Figure 10.** Total Ion Chromatogram (TIC) of UPLC-MS/MS analysis in negative ion mode, showing the overall intensity of all detected ions across the retention time.


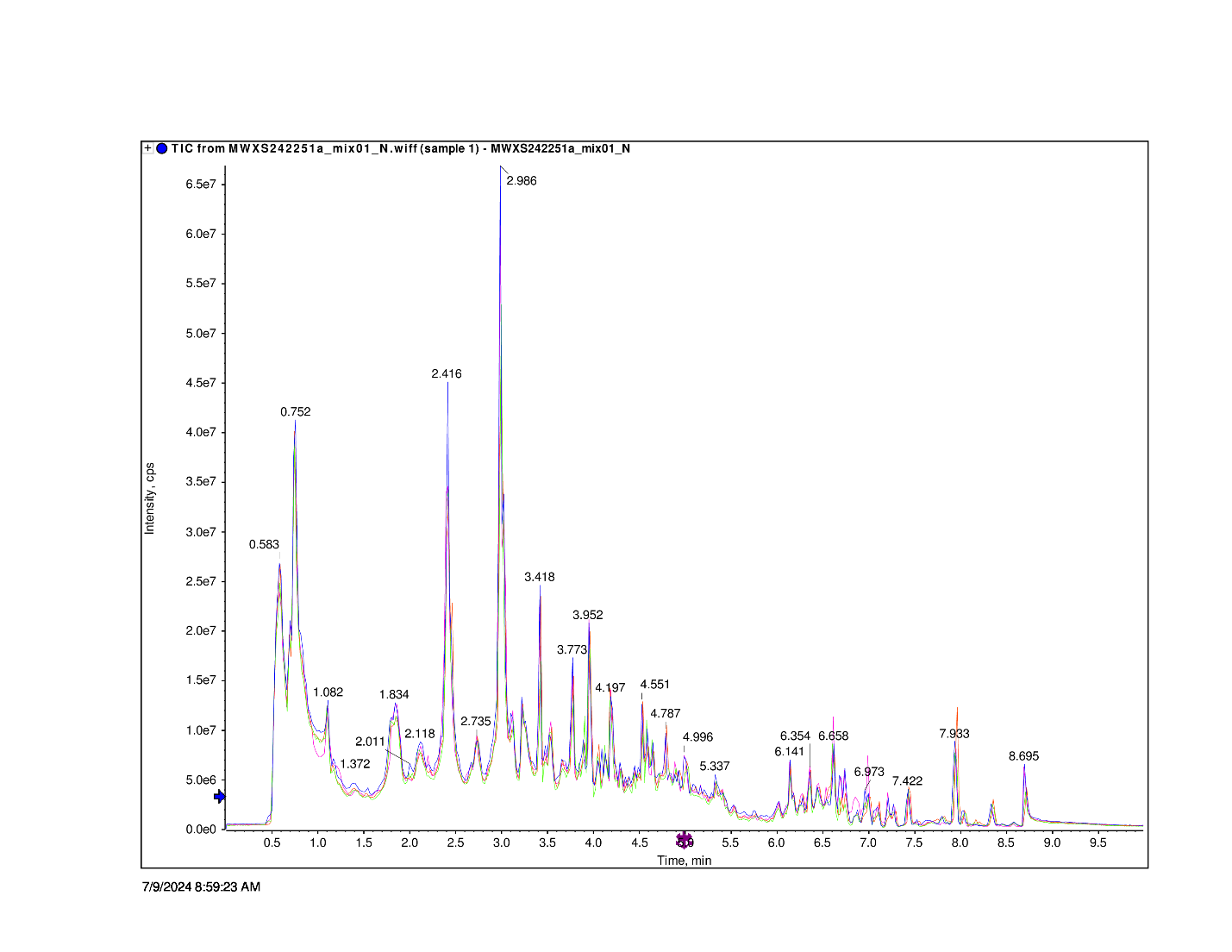


**Supplementary Figure 11.** Extracted Ion Chromatogram (EIC) of UPLC-MS/MS analysis in positive ion mode, showing the retention times and peak intensities of targeted ions in the sample.
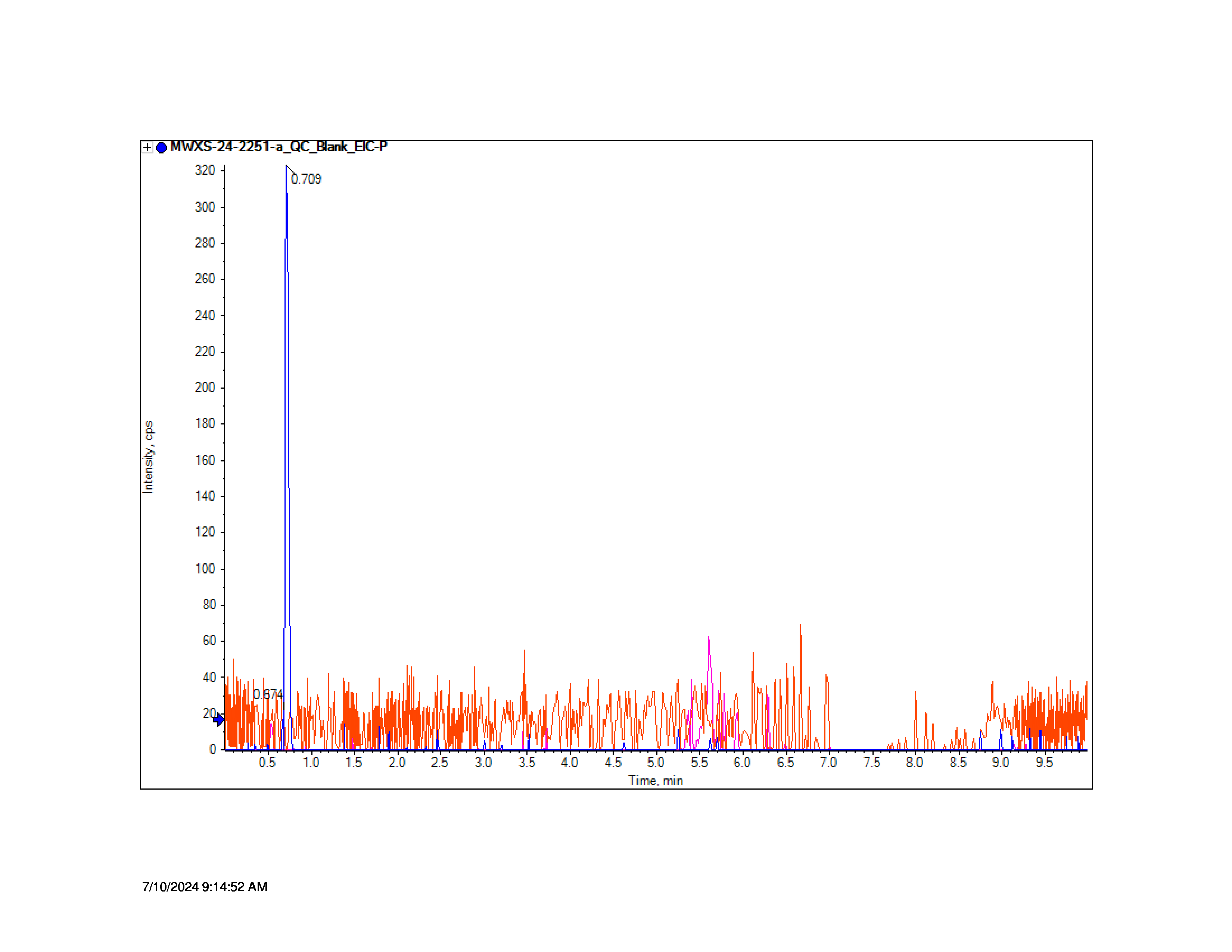


**Supplementary Figure 12.** Total Ion Chromatogram (TIC) of UPLC-MS/MS analysis in positive ion mode, showing the overall intensity of all detected ions across the retention time.


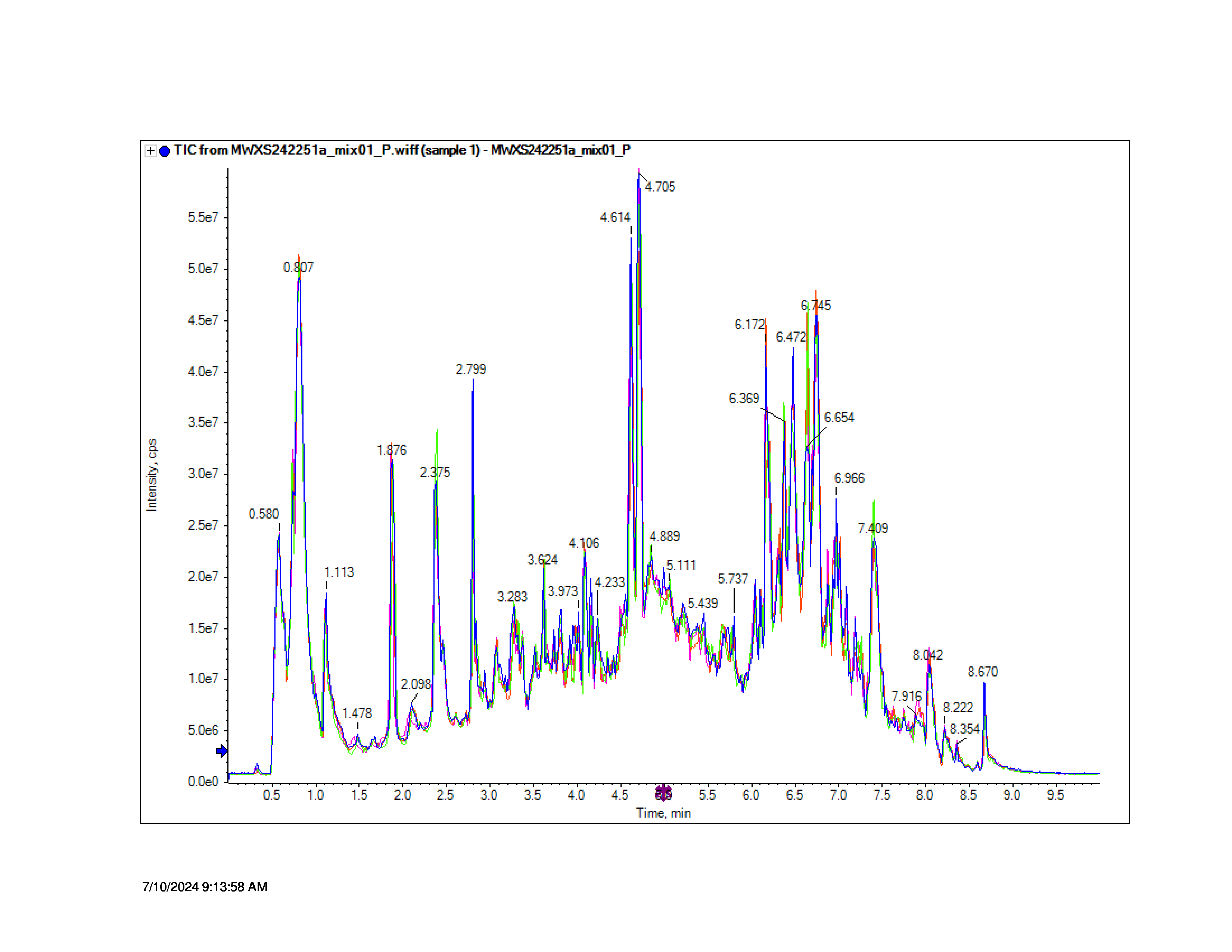


**Supplementary Figure 13.** HCA heat map with dendrogram of *Ferula* species generated using UPLC-MS/MS data, illustrating the clustering patterns and relationships among cultivated *Ferula* species based on their metabolomic profiles.

**
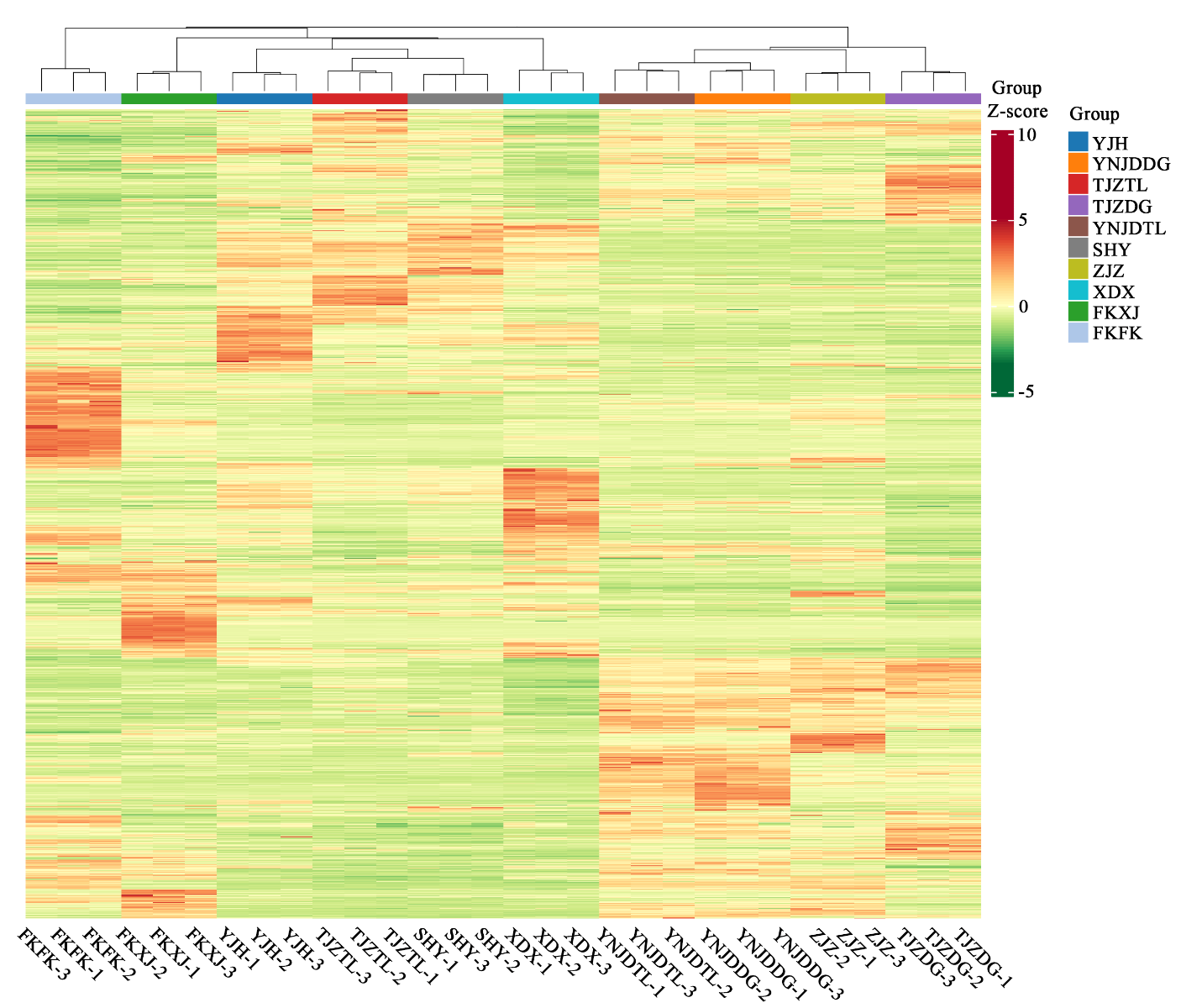
**
